# Synergistic integration of biomaterials in orthopedic implantation for infection preventing and tissue engineering

**DOI:** 10.1093/rb/rbag061

**Published:** 2026-03-24

**Authors:** Longhui Xu, Xu Chen, Zeyang Cao, Guoshuang Zheng, Zhengyong Li, Xiao Yang, Hui Xie, Siyu Chen

**Affiliations:** Department of Orthopaedic, Affiliated Zhongshan Hospital of Dalian University, Dalian 116001, Liaoning Province, China; Department of Orthopaedic, Affiliated Zhongshan Hospital of Dalian University, Dalian 116001, Liaoning Province, China; Department of Orthopaedic, Affiliated Zhongshan Hospital of Dalian University, Dalian 116001, Liaoning Province, China; Central Lab, Affiliated Zhongshan Hospital of Dalian University, Dalian 116001, Liaoning Province, China; Department of Burn and Plastic Surgery, West China School of Medicine, West China Hospital, Sichuan University, Chengdu 610041, China; National Engineering Research Center for Biomaterials, Sichuan University, Chengdu 610064, China; College of Biomedical Engineering, Sichuan University, Chengdu 610064, China; Department of Orthopaedic, Affiliated Zhongshan Hospital of Dalian University, Dalian 116001, Liaoning Province, China; Department of Burn and Plastic Surgery, West China School of Medicine, West China Hospital, Sichuan University, Chengdu 610041, China

**Keywords:** biocomposites, infected bone defects, bone tissue engineering, antibacterial, osteogenesis

## Abstract

The treatment of infected bone defects (IBD) is challenging in orthopedics because of their complex pathological mechanisms and high recurrence rates. An ideal therapeutic strategy should simultaneously eradicate infection and promote bone regeneration. However, conventional approaches rarely achieve both objectives effectively. It has concurrently high recurrence rates, prolonged treatment courses and limited therapeutic efficacy. Clinically, implant-associated infections leading to IBD are often exacerbated by patients’underlying diseases, material-related risks and perioperative factors, thereby necessitating multifunctional strategies to achieve an integrated solution. With the rapid development of bone graft materials, researchers have focused on biocomposites because of their unique multifunctionality. By integrating the advantages of different materials and incorporating various bioactive components, biocomposites allow comprehensive treatment of patients with IBD. In this article, we reviewed the advancements in the application of different types of biocomposites in treating IBD, highlighting their synergistic effects in promoting bone healing and combating infections through various mechanisms. Additionally, it addresses the limitations associated with clinical translation and proposes a regulatory science-based framework for full lifecycle assessment. This framework offers a standardized evaluation system and precise regulatory strategies for personalized biocomposites with multiple functions. These insights provide scientific support and reveal novel approaches for clinical applications.

## Graphical Abstract

**Graphical Abstract:**
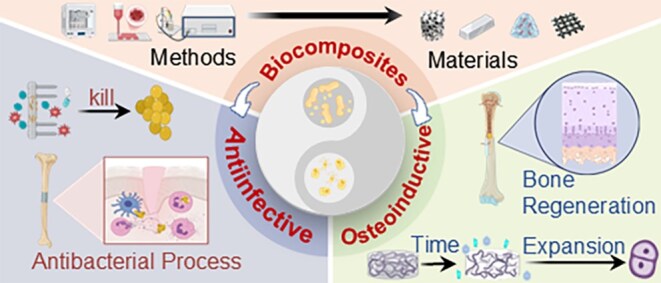

## Introduction

Infectious bone defects (IBD) are pathological conditions characterized by localized or extensive bone destruction caused by microbial invasion, accompanied by persistent inflammation, the formation of necrotic bone and impaired bone regeneration. The treatment of IBD is complex and is quite a challenging problem in orthopedics. IBD is often associated with chronic infection. Sustained local inflammation disrupts the regenerative microenvironment of bone tissue, markedly impairing bone repair and frequently necessitating repeated debridement surgeries to eliminate infectious foci. Moreover, treatment is typically prolonged and associated with frequent complications. Untreated bone infections can develop into systemic infections, becoming life-threatening and affecting society [1]. The treatment of IBD involves infection control and local bone defect reconstruction, where infection control is a prerequisite for reconstruction. The clinical strategies currently used for infection management include debridement of the infected site, systemic administration of antibiotics and implantation of antibiotic-impregnated fillers [2]. However, chronic infections often lead to biofilm formation. Biofilms reduce local blood supply and hinder antibiotics from reaching effective concentrations at the infection site, thereby making pathogen eradication difficult [3, 4]. Thus, prolonged administration of high doses of antibiotics is needed for clinical treatment, which may lead to antibiotic resistance and various complications [5]. Antibiotic-impregnated fillers can deliver high local concentrations of antibiotics, thereby reducing systemic side effects. However, these fillers usually require a second surgery for removal after the initial operation. This two-stage surgical approach increases surgical risk, recovery time and treatment costs [1].

Bone tissue reconstruction can proceed only after the local infection is fully controlled. Common clinical treatments used for managing IBD include bone grafting, the Ilizarov technique and the Masquelet technique [6]. Among these methods, bone grafting is the most important clinical method for treating IBD. However, other methods have limitations, such as prolonged treatment duration, suboptimal outcomes and recurrent infections. In the United States, about 500 000 patients undergo bone grafting annually, with total costs reaching billions of dollars and this number is ever-increasing [7]. Bone grafting materials primarily include autografts, allografts and synthetic bone substitutes [8]. Autografts, regarded as the ‘gold standard’ for bone grafting because of their relatively low degree of immune rejection and risk of infection, often yield excellent osteogenic outcomes [9]. However, autografts have several limitations, including the need for a second surgery, the limited availability of donors and graft resorption. Allografts also pose challenges, for example, immune rejection and poor prognosis.

With the rapid development of bone tissue engineering, artificial bone grafts, which are more effective, safer and resource-efficient functional materials, have become the primary source of materials for bone grafting [10]. Biomedical materials used in bone grafting include inorganic minerals, polymers and metals [11]. Owing to their wide range of sources, these synthetic bone graft materials exhibit diverse structural, chemical and mechanical properties, making them widely accepted for bone reconstruction. However, most of them have limited osteogenic capacity and a poor ability to induce new bone formation in a fibrotic microenvironment [12]. Furthermore, many bone graft materials lack antimicrobial properties and may become potential carriers of infection to surrounding tissues [13].

However, the persistent challenges in treating IBD with artificial bone grafting materials arise not only from the functional limitations of conventional implants but, more critically, from the interaction of multiple complex factors, including patients’ underlying disease conditions, potential material-related risks and surgical as well as postoperative management. Collectively, these factors establish a microenvironment that favors microbial persistence and hampers bone regeneration. A comprehensive investigation of the multifactorial causes of postoperative infections associated with artificial bone implants is, therefore, essential for the development of effective preventive and therapeutic strategies.

Patients’ preexisting disease conditions form an intrinsic basis for infection. For example, individuals with a prolonged history of diabetes often remain in a chronic hyperglycemic state, which not only damages microvascular circulation—resulting in inadequate tissue perfusion and hypoxia—but also impairs the chemotactic and phagocytic activity of immune cells. Concurrently, markedly elevated blood glucose levels provide a nutrient-rich environment that promotes bacterial proliferation [14]. In patients with immune system disorders, or in those receiving immunosuppressive therapy, both innate and adaptive immunity are impaired to varying degrees, markedly reducing the ability to eliminate invading pathogens. As a result, even low-virulence organisms can cause severe and potentially catastrophic infections [15].

Patients with chronic kidney disease often experience toxin accumulation, malnutrition and disturbances in calcium–phosphorus metabolism. These factors directly impair bone quality while also contributing to immune dysfunction and persistent inflammation, which further weaken host defenses. In addition, a history of infection at a previously operated site commonly indicates local tissue scarring, compromised blood supply and the possible presence of dormant bacterial microcolonies. These latent ‘seeds’ can be readily reactivated by foreign implants and surgical trauma, resulting in recurrent infections.

The physicochemical characteristics of implants themselves also pose significant risks of infection. In clinical practice, some conventional inert metallic materials, although mechanically strong, lack intrinsic antibacterial activity and sufficient bioactivity to inhibit pathogen adhesion and growth. During surgery, implant surfaces may be contaminated by bacteria; because of the inert nature of these materials, they cannot directly eliminate bacteria or stimulate immune responses to recruit host defenses for bacterial clearance, thereby greatly increasing the likelihood of postoperative infection. Moreover, wear, corrosion or fatigue of implants during long-term use can release micronscale and nanoscale debris, which trigger chronic inflammatory reactions, continuously stimulate osteoclast activity and accelerate bone resorption. These particles can also adsorb bacteria, acting as carriers and protective niches for bacterial colonization [16]. In addition, regions of surface micro-roughness, manufacturing defects or interfacial gaps on implants are highly prone to bacterial adhesion, creating niches for colonization. Once biofilms develop, bacterial resistance increases substantially, leading to persistent and difficult-to-eliminate infections [17].

In addition, oversights in surgical technique and postoperative care are also critical contributors to infection. Prolonged operative time extends wound exposure, thereby increasing the likelihood of contamination, while persistent mechanical traction and soft tissue injury during surgery can intensify the likelihood of local ischemia–reperfusion injury, triggering inflammatory cascades [18]. Inadequate intraoperative hemostasis or ineffective drainage often leads to the formation of dead space, hematomas or fluid accumulation—conditions that create hypoxic, nutrient-rich microenvironments favorable for bacterial growth. The iron ions abundantly present in hematomas can further promote infection by inducing the expression of virulence factors in certain pathogens [19, 20]. During the early postoperative phase, excessive physical activity may result in implant micromotion or increased wound tension, both of which can disrupt tissue healing. Conversely, prolonged immobilization may cause local blood stasis, thereby impairing the delivery of immune cells and antimicrobial agents to the affected site. Additional factors—such as improper timing of prophylactic antibiotic administration, inadequate treatment duration, failure to target relevant pathogens and substandard wound management—can further exacerbate the risk of postoperative infections following bone implantation [21].

Therefore, developing multifunctional bone graft materials with antibacterial, osteogenic and degradable properties to facilitate one-stage treatment of IBD, thereby avoiding multiple surgeries holds significant clinical value. Biocomposites integrate the advantages of various materials, substantially improving material performance while offering multifunctionality and customization. This makes biocomposites suitable for bone grafting applications, and they show significant potential in tissue-engineered treatments for IBD. Most existing reviews are limited to single materials or specific treatment mechanisms and lack a comprehensive exploration of multifunctional composites. This review combines the design principles of biocomposites and focuses on advanced technologies to optimize the preparation and clinical application of multifunctional materials. This review discusses the multifunctional synergistic effects of different biocomposites in treating IBD, providing new insights into their treatment. Additionally, this review proposes a life-cycle assessment framework, focusing on the entire process from the laboratory to clinical application, filling the gap in previous studies regarding the clinical assessment system for biocomposites.

## Pathology and therapeutic challenges associated with IBD

IBD are generally caused by bacteria, with common pathogens, including *Staphylococcus aureus* (*S. aureus*), *Escherichia coli* (*E. coli*), *Staphylococcus epidermidis* (*S. epidermidis*), *Streptococcus* spp. [22]. These bacteria penetrate the body’s defense barriers through trauma, implants and diseases, invading the deep skeletal system [23]. Pathogenic bacteria not only directly impair bone regeneration but also indirectly influence the osteogenic process by altering the microenvironment [24]. After invading the bone and adhering to bone matrix components, bacteria secrete pathogenic factors that disrupt physiological homeostasis, directly induces osteoblast apoptosis and exacerbates inflammatory responses, leading to the rapid release of proinflammatory factors. Persistent inflammation results in excessive activation of osteoclasts and inhibition of osteogenesis, ultimately resulting in pathological bone resorption. Owing to the lack of blood supply and metabolic activity, necrotic bones lose their mechanical properties and repair capacity, ultimately resulting in irreparable IBD [25]. The reported pathological mechanisms affecting IBD mainly include the following factors:

### Changes in the inflammatory microenvironment

**Figure 1:**
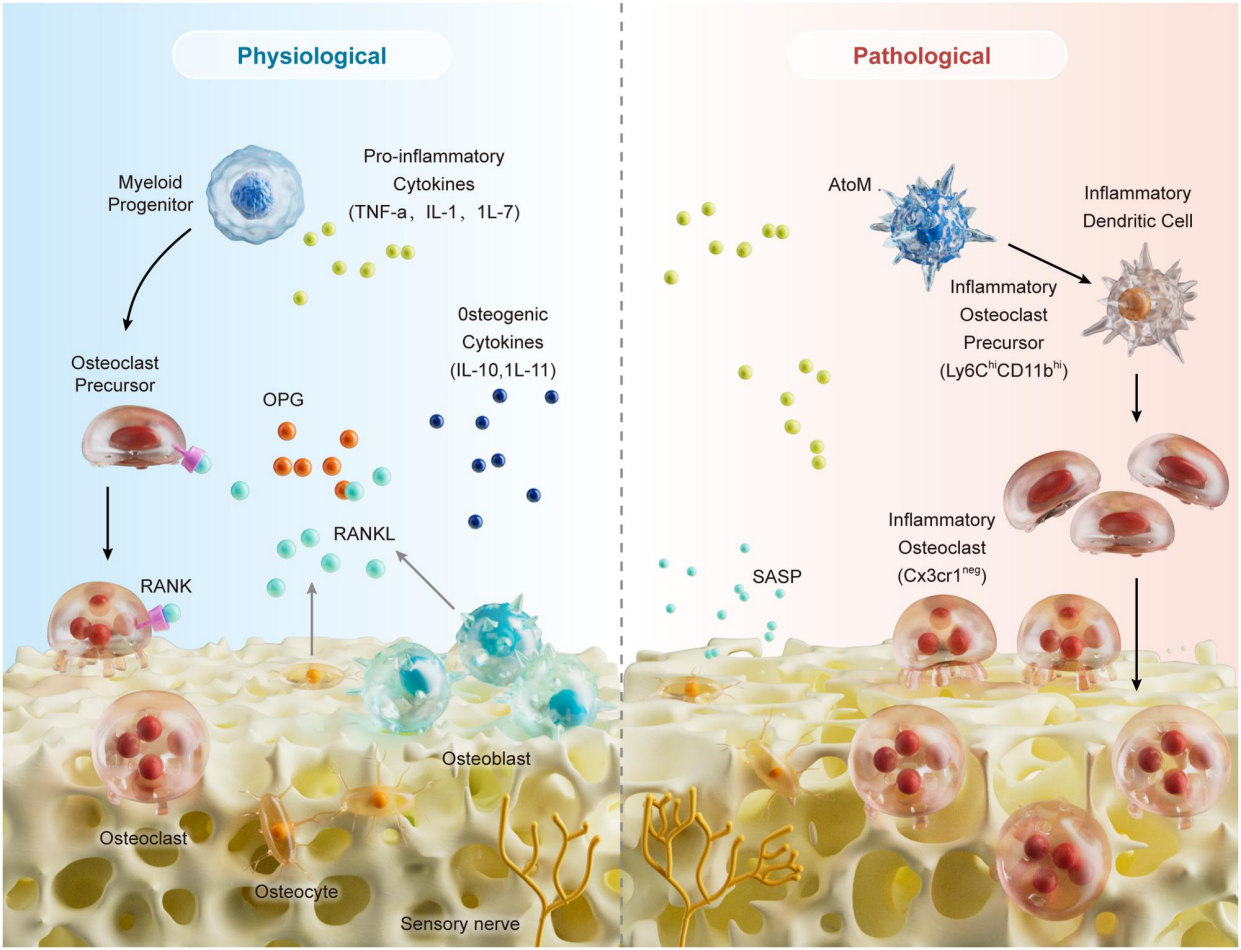
The impact of inflammatory processes in physiological and pathological conditions on bone repair.

Bone repair is initiated by an inflammatory response, and a controlled and moderate level of inflammation is necessary for subsequent bone reconstruction. However, when infection serves as a persistent stimulus, it leads to sustained excessive inflammation at the site of bone injury, resulting in fibrosis and various complications that hinder bone repair (Figure 1) [26]. Compared with those in aseptic bone injury, the levels of inflammatory cells, for example, helper T cells, cytotoxic T cells and proinflammatory factors, such as interleukin (IL)-1β, are significantly greater in IBD [27]. Activated T cells and B cells release large amounts of receptor activator of nuclear factor kappa-B ligand (RANKL), which induces bone resorption [28]. Inflammatory cells, for example, macrophages and T cells, secrete various proinflammatory cytokines and mediators that promote osteoclastogenesis through RANKL-dependent and RANKL-independent pathways [29]. Excessive bone resorption disrupts the balance between osteoblasts and osteoclasts, impairing bone repair. In addition, numerous studies have revealed that certain proinflammatory factors can directly inhibit the process of osteogenic differentiation. Lacey *et al*. [30] observed that IL-1β can inhibit the proliferation of mesenchymal stem cells (MSCs) *in vitro*, reduce the expression of the key osteogenic gene Runx2 and alkaline phosphatase, and affect the mineralization of the extracellular matrix. Liu *et al*. [31] demonstrated that interferon-γ can reduce the expression level of the key osteogenic gene RUNX-2.

### Damage to the extracellular matrix

**Figure 2:**
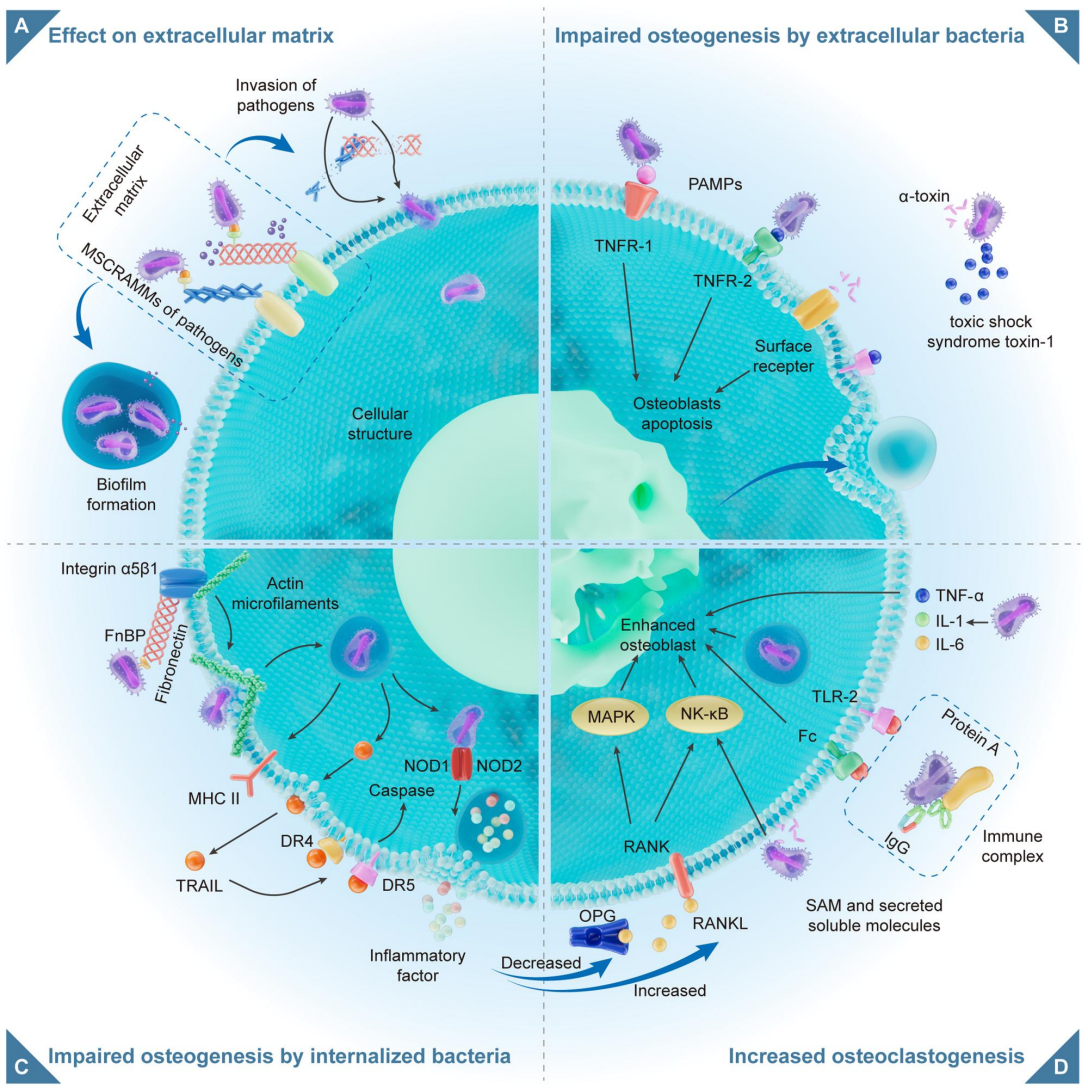
Four negative mechanisms of infection affect bone repair. (**A**) Pathogens bind to the extracellular matrix (ECM) via MSCRAMMs on their surface, degrading ECM components and forming biofilms that hinder tissue repair; (**B**) Pathogens or their pathogen-associated molecular patterns (PAMPs) bind to osteoblast surface receptors (e.g. TNFR-1 and TLR-2), activating apoptotic signals and secreting toxins (e.g. alpha-toxin), leading to the apoptosis of osteoblasts; (**C**) Pathogens enter osteoblasts via the interaction of integrins and fibronectin, after which they disrupt the cytoskeleton, activate apoptotic pathways and secrete proinflammatory factors, sustaining excessive inflammation; (**D**) Infection-induced excessive inflammation increases the expression of proinflammatory cytokines (e.g. TNF-α, IL-1 and IL-6), enhances RANKL expression, reduces the expression of OPG and promotes osteoclastogenesis and activity, thereby increasing bone resorption and impairing bone repair.

In bone tissue, interactions between the extracellular matrix (ECM) and cell surface receptors or adhesion molecules play key roles in regulating cell differentiation and maturation [2]. When pathogens invade the bone defect area, their surface adhesion molecules facilitate tight binding to the bone ECM (Figure 2A). After binding to the bone ECM and colonizing, pathogens subsequently produce various virulence factors that degrade ECM components, further facilitating bacterial invasion [32, 33]. The interaction between ECM components and osteoblast surface receptors is also a key signaling pathway that regulates the differentiation, maturation and mineralization of osteoblasts [34, 35]. Therefore, damage to the ECM reduces local bone repair capacity.

During chronic infection, the ECM integrates with biofilms, forming biofilms containing dense bacterial colonies. Biofilm formation helps bacteria resist antibiotic treatment and immune system attacks, thereby decreasing the effectiveness of antibacterial therapy [36]. Additionally, biofilms serve as bacterial reservoirs where bacteria can survive and disperse from mature biofilms, exacerbating infection [37].

### Inhibition of osteoblasts

Pathogens directly bind to pattern recognition receptors (PRRs) on the surface of osteoblasts via their surface pathogen-associated molecular patterns (PAMPs), for example, endotoxins and flagellar proteins, thereby initiating an immune response (Figure 2B) [38]. These PAMPs can bind to receptors, such as Toll-like receptors (TLRs), tumor necrosis factor receptor 1 (TNFR-1) and nucleotide-binding oligomeric structure-related proteins (NLRs), thereby inducing osteoblast apoptosis and inhibiting their differentiation. For illustration, TLR-2 and TNFR-1 are extracellular receptors that can recognize PAMPs from *S. aureus*, leading to osteoblast apoptosis, and thereby affect bone repair [39].

Pathogen invasion of osteoblasts is another important factor affecting bone regeneration [40]. Osteoblast infections occur through multiple pathways and are initiated by interactions between microorganisms and ECM components. For instance, during *S. aureus* infection, fibronectin in the ECM binds to bacterial fibronectin-binding proteins while simultaneously interacting with the osteoblast surface integrin α5β1, forming an indirect connection between *S. aureus* and osteoblasts (Figure 2C) [24, 41]. The internalization of bacteria in osteoblasts is an active process that requires the participation of cytoskeletal components, for example, actin filaments and microtubules [42]. Once internalized, *S. aureus* undergoes phenotypic changes, transforming into a low-sensitivity subtype, which enables long-term survival within osteoblasts. These changes allow the fungus to evade immune clearance and complicate antibiotic treatment. Consequently, infected osteoblasts may serve as bacterial reservoirs, continuously releasing bacteria to infect other cells [42, 43].

### Promotion of osteoclasts

The balance between osteoblasts (bone regeneration) and osteoclasts (bone resorption) is crucial for bone repair [44]. In the microenvironment in which IBD occur, pathogens induce an increase in osteoclasts. Osteoclasts originate from hematopoietic monocytes/macrophages and are driven by macrophage colony-stimulating factor (M-CSF) and receptor activator of RANKL. M-CSF and RANKL promote the proliferation and maturation of osteoclast precursors, respectively, and play key roles in osteoclast activation [45]. During infection, pathogens directly promote the secretion of RANKL by infecting osteoblasts and downregulating the expression of osteoprotegerin (OPG), thereby increasing the production of osteoclasts (Figure 2D) [46]. OPG is secreted by osteoblasts and MSCs and serves as a decoy receptor for RANKL, which negatively regulates the differentiation of osteoclasts [47].

The surface-associated molecules (SAMs) of bacteria and their secreted soluble molecules can directly promote the differentiation of osteoclasts, along with their indirect effects on osteoclast differentiation via osteoblasts. Studies have shown that the SAMs of *S. aureus* directly promote osteoclast formation through a RANKL-independent pathway [48, 49]. Unlike osteoblast infection, the internalization of *S. aureus* by osteoclasts may occur through phagocytosis. Osteoclasts cannot prevent the internalization of *S. aureus* but can promote osteoclast fusion and promote bone resorption activity [50, 51].

## Current status of design and application of biocomposites

The emergence of biocomposites as third-generation bone graft materials represents a significant milestone in the development of bone graft materials. Biocomposites are multifunctional materials composed of two or more different materials, created through physical or chemical methods (Table 1). They are designed to enhance the performance of a single material or endow it with new biological functions. They are widely used in human tissue repair and functional reconstruction [52]. These materials not only have excellent mechanical properties and bioactivity but also incorporate various functional components, such as antibacterial agents, growth factors and gene carriers, providing comprehensive therapeutic effects. After they are implanted into defect sites, they offer necessary mechanical support and bioactivity to promote bone regeneration, while the sustained release of antibacterial agents effectively controls and prevents infection. With continuous technological advancements, including nanotechnology and 3D printing, the design and fabrication of biocomposites have become more precise and personalized, catering to the specific needs of different patients. The use of biocomposites is a promising approach for treating IBD and is a key research focus.

**Table 1:** Material selection for designing biocomposites.

Material classification	Material name	Application	Advantages	Disadvantages	References
Inorganic minerals	HA	Bone repair scaffold, often combined with CS or PLGA to enhance mechanical properties and antibacterial performance.	• Strong biocompatibility • Osteoconductivity	• Poor mechanical properties	[74–77]
	Bioactive glass (BG)	Infection control and bone regeneration, can be used alone or in combination with antibacterial agents to enhance anti-biofilm capability.	• Promotes bone regeneration • Robust antibacterial activity	• Suboptimal degradation rate	[78, 79]
Natural polymers	Alginate	Drug delivery carrier or 3D-printed scaffold, providing three-dimensional cell adhesion and bone regeneration support.	• Excellent biocompatibility • Good gelation properties	• Inadequate mechanical properties • Structural instability	[80]
	Chitosan	Commonly used as a matrix for HA or BG, to prepare multifunctional composites for enhanced antibacterial and osteogenic capabilities.	• Strong antibacterial properties • Good osteogenic activity	• Limited antibacterial capacity • Poor solubility	[81–83]
Synthetic polymers	PCL	3D-printed complex bone defect scaffolds, providing long-term bone support and osteogenesis promotion.	• Strong mechanical properties • Good toughness	• Slow degradation rate • Inadequate mechanical properties	[84, 85]
	PLGA	Repair of IBD, serving as a drug carrier or bone scaffold to improve osteogenic efficiency.	• Controllable degradation • Good mechanical properties	• Inadequate mechanical properties • Insufficient bioactivity	[86]
	PMMA	PMMA is widely employed in orthopedics and dentistry, most notably as bone cement in joint replacement surgeries, as well as in drug delivery systems.	• Excellent mechanical strength • High moldability • Strong antibacterial properties	• Elevated temperatures may compromise antibiotic activity • Lack of biodegradability	[87–89]
	PLA	PLA is primarily utilized in bone repair scaffolds, 3D-printed materials for osseous defects and drug delivery applications.	• Biodegradable • Excellent biocompatibility	• Relatively low mechanical strength • Degradation rate difficult-to-control	[90–92]
Metal materials	Stainless Steel (SS)	Orthopedic implants, combined with antibacterial coatings to reduce infection risk.	• Superior mechanical strength	• Poor corrosion resistance	[93–95]
	Cobalt (Co)	Load-bearing bone repair implants.	• High corrosion resistance • Good mechanical properties	• High elastic modulus • High cost • Weak antibacterial activity	[96]
	Titanium (Ti)	Bone fixation and repair implants, with surface coatings to enhance antibacterial and bone induction performance.	• Excellent mechanical properties • High corrosion resistance	• High elastic modulus • Potential cytotoxicity	[97–99]
	Magnesium (Mg)	Bone repair of IBD, with natural degradation after implantation to avoid secondary surgery.	• Excellent biodegradability • Promotes bone regeneration	• Rapid degradation accompanied by hydrogen release	[100, 101]
	Tantalum (Ta)	Repair of complex bone defects, with 3D printing to create porous structures that promote bone ingrowth.	• Excellent mechanical properties • Good osteogenic ability	• High cost • Significant processing difficulty	[102, 103]
Nanomaterials	Carbon Dots	widely applied in bone tissue imaging and drug delivery systems and can also serve as a carrier for antioxidants and anti-inflammatory drugs.	• Excellent fluorescence labelling capability • Good antioxidant capacity • Good anti-inflammatory ability	• Low drug-loading capacity • Limited bioactivity	[104, 105]
	Graphene	Graphene and its derivatives are widely used in bone repair, drug delivery and biosensor fields.	• Excellent mechanical properties • Excellent conductivity	• Poor degradability • Potential toxicity concerns	[106–108]

In this review, the term ‘synergy’ does not denote a simple combination of different materials; rather, it specifically refers to functional synergy. Within a single biocomposites system, key functions—including antibacterial activity, osteogenesis and modulation of host responses—are temporally and spatially integrated to overcome the intrinsic trade-offs commonly encountered in single-function designs. The objective is to achieve effective antibacterial performance while minimizing cytotoxicity, and to promote osseointegration while simultaneously reducing the risk of infection recurrence. Therefore, the criterion for synergy is not merely the additive increase in the number of functions, but whether the composite system attains a superior overall balance between therapeutic efficacy and biosafety. In essence, synergy should be evaluated based on the system-level optimization of functional outcomes rather than on functional accumulation alone.

Bone tissue requires materials that provide sufficient mechanical strength to withstand physiological loads and provide stable support, especially during the repair process of weight-bearing areas, for example, long bones. The mechanical properties of natural bone vary depending on its location. For example, the compressive modulus of cortical bone is generally between 17 and 20 GPa, whereas that of cancellous bone is about 0.1–0.5 GPa [53]. Therefore, the mechanical properties of biocomposites should closely match those of bones to prevent premature degradation or deformation after implantation. Additionally, the elastic modulus of the material should be compatible with that of natural bone to avoid the ‘stress shielding’ effect; an overly rigid implant reduces the load on surrounding natural bone tissue, thereby inhibiting the growth of new bones. The elastic modulus of the material should be adjusted according to the specific application. The stiffness and strength of a material influence the mechanical stability of bones and regulate cell behavior through mechanotransduction, promoting osteoblast differentiation and deposition in the bone matrix [54]. Beyond structural support, early mechanical stability is an indispensable prerequisite for the management of IBD. Even micromotion and interfacial instability can lead to exudation, sustained inflammation and persistent bacterial colonization, thereby compromising both infection control and osseointegration [55].

With respect to functional properties, biocompatibility is a fundamental requirement for all biomaterials. The material must bind well to the host tissue and support cell adhesion, proliferation and differentiation without triggering an immune rejection response. Natural polymers such as collagen (COL), chitosan (CS) and hyaluronic acid perform well in terms of biocompatibility because of their similarity to the body’s tissues. Moreover, these materials can also promote functional responses in cells by regulating biological signals in the extracellular matrix [56, 57]. However, although some synthetic polymers, such as polylactic acid (PLA), polyglycolic acid (PGA) and polycaprolactone (PCL), exhibit good mechanical properties and degradability, they have certain limitations in terms of biocompatibility and are prone to causing local inflammatory reactions [58]. Therefore, modern biocomposites combine the advantages of both natural and synthetic polymers to ensure biocompatibility, adequate stability and mechanical strength *in vivo*. To further enhance biocompatibility, researchers often use surface modifications or incorporate osteogenic bioactive factors to improve the activity of osteoblasts and accelerate bone tissue regeneration. It is worth emphasizing that, in the repair of IBD, biocompatibility should not be narrowly interpreted as merely the absence of immune rejection. An ideal biocomposite should actively modulate the host immune response toward a pro-regenerative phenotype, preserving essential anti-infective defense mechanisms while attenuating excessive or chronic inflammation [59]. Therefore, osteoimmunomodulation plays a critical bridging role between antibacterial function and osteogenic regeneration, and constitutes an indispensable component in achieving functional synergy.

The biodegradability of biomaterials is also a crucial characteristic that cannot be overlooked in the process of bone regeneration. Biodegradability refers to the ability of a material to dissolve over time when it is implanted into the body, accompanied by a decrease in the mechanical properties of the implanted material [60]. The ideal material should gradually degrade within the body at a rate that matches the growth rate of new bone tissue. Too rapid degradation may cause the implant to lose its supportive function, whereas too slow degradation may hinder the formation of new bone. In practical applications, the degradation rate of a material can be achieved by regulating its composition, preparation process and microstructural characteristics. For example, synthetic polymers such as PLA and PCL have relatively slow degradation rates and are typically used for the long-term repair of bone defects, whereas natural polymers, for example, CS and hyaluronic acid, have relatively fast degradation rates and are suitable for short-term repair. To date, most research has focused on understanding how the physiological environment alters materials, but it is equally important to study how surrounding cells respond to degrading materials. Material degradation is not merely a process of structural evolution, but also a critical determinant of microenvironmental regulation. The degradation products and their release kinetics can markedly influence local pH, ionic composition and inflammatory signaling pathways, thereby simultaneously modulating bacterial activity, biofilm formation and the behavior of osteogenic and immune cells [61]. Therefore, a degradation profile tailored to match the dynamics of bone regeneration represents an essential regulatory strategy for achieving synergistic integration of antibacterial and osteogenic functions.

Osteoinductivity is a crucial function of biocomposites, which require both good osteoconductivity and osteoinductivity. Bone conduction refers to the ability of a material to provide a scaffold for the attachment, migration and growth of new bone. Many inorganic materials, such as hydroxyapatite (HA) and calcium phosphate (CaP), exhibit good osteoconductivity, promoting new bone deposition and maturation. By combining these inorganic materials with natural or synthetic polymers, the bone conduction properties of the materials can be further enhanced, allowing bone cells to adhere to and proliferate on the surface and form new bone tissue [62]. Moreover, these inorganic components can activate the differentiation pathway of osteoblasts by releasing osteogenic factors, for example, Ca^2+^, thereby enhancing the deposition of the bone matrix [63]. Bone induction refers to the ability of a material to induce undifferentiated MSCs to differentiate into osteoblasts, a process that is typically achieved through the addition of growth factors [64]. In recent years, researchers have attempted to combine various growth factors, drugs or genetically engineered products into biomaterials to promote bone regeneration; for instance, bone morphogenetic protein-2 (BMP-2) and vascular endothelial growth factor (VEGF) are often used to stimulate osteoblast differentiation and new blood vessel formation [65]. In addition, ECM components, such as COL and hyaluronic acid, are often used as the matrix of biocomposites to provide the necessary biochemical signals to support bone cell migration and function. Moreover, the surface roughness of a material affects cell adhesion and osteogenic capacity. A rough surface can promote osteoblast adhesion and proliferation by providing more attachment points [66]. During the treatment of IBD, the efficacy of osteogenesis is highly dependent on the stability and controllability of the local microenvironment. Infection and the accompanying inflammatory response often exert sustained inhibitory effects on osteoblast adhesion, differentiation and matrix deposition [67]. Meanwhile, if antibacterial strategies lack precise regulation, the resulting cytotoxicity or amplification of inflammatory responses may also adversely affect the osteogenic process. In this context, osteogenic function does not operate independently, but can only be fully realized under the support of effective infection control and immune modulation.

In the repair of IBD, anti-infective properties are also a key consideration in material design. One of the main challenges of treating IBD is that the presence of bacteria can lead to biofilm formation, which hinders antibiotic penetration and makes it difficult-to-eradicate the infection. Therefore, ideal biocomposites should possess antibacterial properties or be able to bind antibiotics to enhance their anti-infective capabilities. For illustration, CS, a natural antibacterial material, is often used in biocomposites. It not only prevents the spread of infection by inhibiting bacterial growth but also promotes tissue repair by regulating the local microenvironment [68]. In addition, the development of nanotechnology in recent years has provided new ideas for the design of antimicrobial materials. Research has shown that nanomaterials, for example, AgNPs, ZnO and TiO^2^, have significant advantages in terms of antibacterial properties and can effectively inhibit the growth of a wide range of gram-positive and gram-negative bacteria. For instance, AgNPs can generate reactive oxygen species (ROS), destroy bacterial cell membranes and organelles and induce cell death. In addition, Ag^+^ can also bind to sulfhydryl groups in bacterial proteins, interfering with their metabolic processes [69]. Moreover, the addition of these nanomaterials can also enhance the mechanical properties and bioactivity of biocomposites, further improving their application prospects in bone defect repair [70]. ZnO and TiO^2^ are also commonly used inorganic nanomaterials in biocomposites. ZnO inhibits bacterial growth by releasing Zn^2+^, disrupting the bacterial enzyme system and inducing oxidative stress reactions [71]. TiO^2^ is activated by ultraviolet light to generate large amounts of ROS, which can effectively kill bacteria. This material is particularly suitable for the treatment of open bone defects and can enhance its antibacterial effect under light exposure conditions. However, how to effectively utilize this property within the body remains a technical challenge [72]. However, metals are a double-edged sword, and while they can kill bacteria, they also exhibit cytotoxicity. Antibiotic delivery systems are also an important antimicrobial strategy. By encapsulating antibiotics in porous structures or polymer matrices, controlled drug release can be achieved [73]. This design prevents the spread of infection while avoiding the problem of antibiotic resistance caused by excessive use of antibiotics. Moreover, some drug delivery systems can also be combined with bone regeneration promoters to achieve both antibacterial and bone-promoting effects. To enhance the efficacy of antibacterial strategies, it is essential to consider both the timeliness and sustained release of antimicrobial agents. This ensures that antibacterial activity not only achieves effective bacterial suppression during the early stage of infection but also persists throughout the critical phases of tissue repair, thereby preventing recurrence and avoiding premature re-exposure to infection during bone regeneration. Such a strategy provides a stable physiological foundation for subsequent vascularization and osteogenesis, effectively bridging the temporal window between infection control and tissue regeneration.

In summary, antibacterial activity, osteogenesis, material degradation and immune regulation are not isolated functional modules; rather, they constitute an integrated system through spatiotemporal regulation and functional coupling. At different stages of IBD repair, the emphasis of each functional module varies: during the early phase of infection, antibacterial activity is the primary priority. As the infectious burden decreases, immune modulation and vascularization gradually assume dominant roles; ultimately, osteogenic function completes bone reconstruction under a properly regulated immune environment with adequate vascular support. Through rational design of temporal regulation and spatial distribution within the material system, the effectiveness of each functional module can be enhanced while minimizing the negative interference of any single function on others, thereby maximizing the overall therapeutic outcome.

### Inorganic-based biocomposites

#### HA-based biocomposites

HA is the primary inorganic component of bone and is considered one of the most ideal bone repair materials because of its excellent biocompatibility, bioactivity and osteoconductivity [109]. In the 1980s, the Aoki team in Japan successfully synthesized HA, marking the beginning of its use as a biomaterial and in clinical research [110]. However, HA has poor mechanical properties and lacks antibacterial activity, limiting its application as a bone implant material. Current research on HA-based biocomposites draws inspiration from the structure and chemical composition of natural bone tissue to create biomimetic compounds, such as composites with polymer materials, which are expected to mimic the organic and inorganic parts of natural bone, not only providing support for bone regeneration but also serving as drug delivery carriers [111, 112].

**Figure 3:**
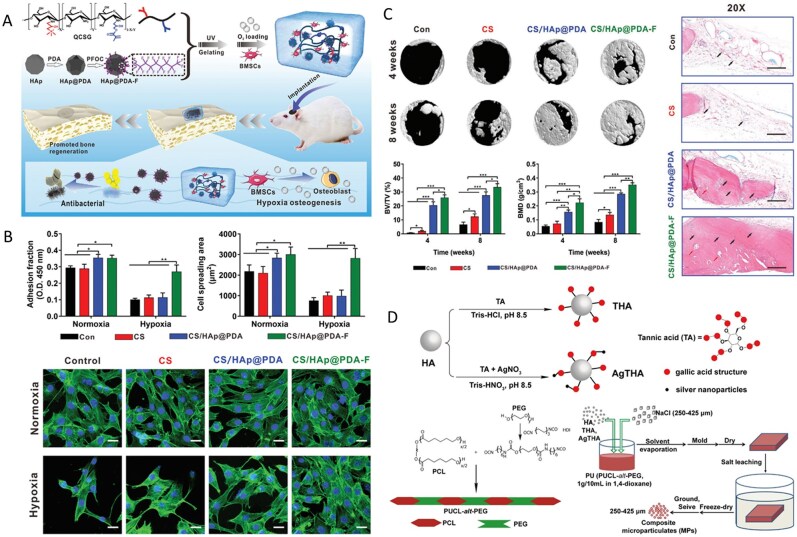
Synthesis and biological evaluations of multifunctional HA-based biocomposites. (**A**) Schematic diagram of composite hydrogel construction. (**B**) The cell adhesion and spreading under normoxia and hypoxia. (**C**) *In vivo* evaluation of bone regeneration. Reproduced with permission from Ref. [117], Copyright 2022, Wiley. (**D**) Synthesis process of hydroxyapatite/polyurethane composite microspheres (PU/Ag-THA). Reproduced with permission from Ref. [119], Copyright 2021, Elsevier.

Sathiyavimal *et al*. [113] developed a mussel shell-derived HA/CS-GA composite scaffold via an *in situ* synthesis method to address the complex pathological challenges of IBD through a dual-function synergistic mechanism. The pH-responsive release of gentamicin effectively inhibits pathogenic bacteria, for example, *S. aureus*, disrupts biofilms and alleviates the inhibitory effects of the inflammatory microenvironment on osteoblasts. Moreover, nanoscale hyaluronic acid and CS form a biomimetic fiber network through hydrogen bonds, simulating the mechanical-chemical microenvironment of the bone ECM, promoting the adhesion and proliferation of MG-63 cells and reversing the osteogenic-osteoclastic imbalance induced by pathogens. This material simultaneously achieves infection control and bone regeneration, precisely addressing the clinical challenges of ‘anti-infection and promotion of repair’ in IBD. The 3-(4,5-dimethylthiazol-2-yl)-2,5-diphenyltetrazolium bromide (MTT) method was used to observe the proliferation of MG63 cells on the surface of HA/CS biocomposites containing different concentrations of CS, revealing that CS has a concentration window in HA/CS biocomposites. Within this concentration window, CS has the dual function of selectively killing bacteria without affecting normal osteoblasts while enhancing their differentiation and proliferation [114]. Additionally, researchers have reported that introducing permanently positively charged quaternary ammonium groups onto the free amino or hydroxyl groups of chitosan can modify it into quaternary ammonium-modified chitosan (HACC), which exhibits improved solubility and stronger antibacterial activity, effectively eliminating drug-resistant bacteria [115]. The antibacterial activity of HACC is positively correlated with the number and proportion of quaternary groups, that is, the degree of substitution. However, an excessively high degree of substitution may cause cytotoxicity [116]. Huang *et al*. [117] developed a multifunctional fluorinated nano-HA composite hydrogel (CS/HA@PDA-F). This hydrogel uses polydopamine as a bridge to embed fluorinated nano-HA (HA@PDA-F) into a quaternized methyl acrylate chitosan matrix. Owing to the high oxygen affinity of fluoride ions, the hydrogel can sustainably release oxygen under hypoxic conditions for up to one week, significantly increasing the survival rate, proliferative activity and osteogenic differentiation potential of MSCs. Moreover, the nonpolarity and rod-shaped conformation of fluoride give the hydrogel broad-spectrum antibacterial properties, with high inhibition rates against *S. aureus* and *E. coli*, and reduce the risk of postoperative infection by reducing bacterial biofilm formation (Figure 3A–C).

Traditional antibiotic treatment of IBD is prone to causing bacterial resistance. Metal ions such as silver ions, which destroy the integrity of bacterial membranes, inhibit DNA replication and produce ROS, are ideal alternatives with broad-spectrum antibacterial properties [118]. Tian *et al*. [119] developed a tannic acid-silver synergistically modified HA/polyurethane composite microsphere (PU/Ag-THA) that achieved synergistic enhancement of antibacterial and bone regeneration properties through multiscale material design (Figure 3D). This material utilizes HA as a carrier and, through tannic acid-mediated reduction and complexation, stably anchors silver nanoparticles (Ag NPs) to the HA surface, combining them with alternating block polyurethane (PUCL-alt-PEG) to construct porous microspheres. The results showed that the material reduced the *in vivo* antibacterial rate against *S. aureus* to less than 3% at 12 weeks postsurgery in a rat model of critical-sized infected femoral ankle joint defects, significantly inhibiting biofilm formation. Additionally, tannic acid delays the release of Ag^+^ through phenolic hydroxyl chelation, preventing the cellular toxicity caused by local concentration spikes, and synergistically enhances antimicrobial persistence. In the infected femoral condyle defect rat model, the bone density of the PU/Ag-THA group at 12 weeks postsurgery was significantly greater than that of the blank control group and showed a sustained growth trend.

For diabetic patients, Yan *et al*. [120] designed a multifunctional composite dressing composed of PLGA-PEG-Ag/Si@HA-SA, designed to combat infection and enhance wound healing. The dressing features a hydrophobic outer layer that minimizes bacterial adhesion and inhibits biofilm development. Simultaneously, it ensures a controlled release of Ag^+^ from the silver/silicon-doped hydroxyapatite component, which exerts potent antibacterial effects by disrupting bacterial respiration and damaging DNA structures. The scaffold also releases ${\text{SiO}}_{4}^{2-}$, which stimulate new blood vessel formation and improve local blood flow—critical for enhancing immune function in diabetic wounds. Moreover, the inner functional layer utilizes a dual-directional biofluid transport system that actively removes excess exudate while recycling Ag^+^ and ${\text{SiO}}_{4}^{2-}$ back into the wound site, collectively reducing inflammation duration and accelerating tissue regeneration. CS-HA nanocomposites, typically synthesized via precipitation, often suffer from the uncontrolled migration of nanoscale HA particles into adjacent tissues, potentially causing cellular damage. To address this, Kong *et al*. [121] developed a strategy to synthesize nano-HA directly within the chitosan matrix through *in situ* chemical methods. This approach facilitated uniform dispersion and tight integration of the HA particles. By employing freeze-drying techniques, they created a porous scaffold architecture that effectively limited HA migration, minimized tissue irritation and exhibited superior biocompatibility compared to conventional CS-based materials. Wojcik *et al*. [122] developed a Zn-HA/curdlan composite wound dressing, wherein zinc-doped hydroxyapatite functions as a slow-release platform for Zn^2+^, offering sustained antimicrobial activity at the surgical site. The dressing’s gel-like porous matrix actively captures bacteria, enabling physical removal during dressing changes and thereby reducing postoperative infection risk. Tested in an animal model of ocular postoperative infection, the implantable dressing served as a bioactive filler that successfully balanced infection control with tissue compatibility. It significantly decreased the need for additional surgical interventions and presents a promising option for managing postoperative wounds in high-risk patients, especially those with diabetes.

#### Mineralized collagen-based biocomposites

**Figure 4:**
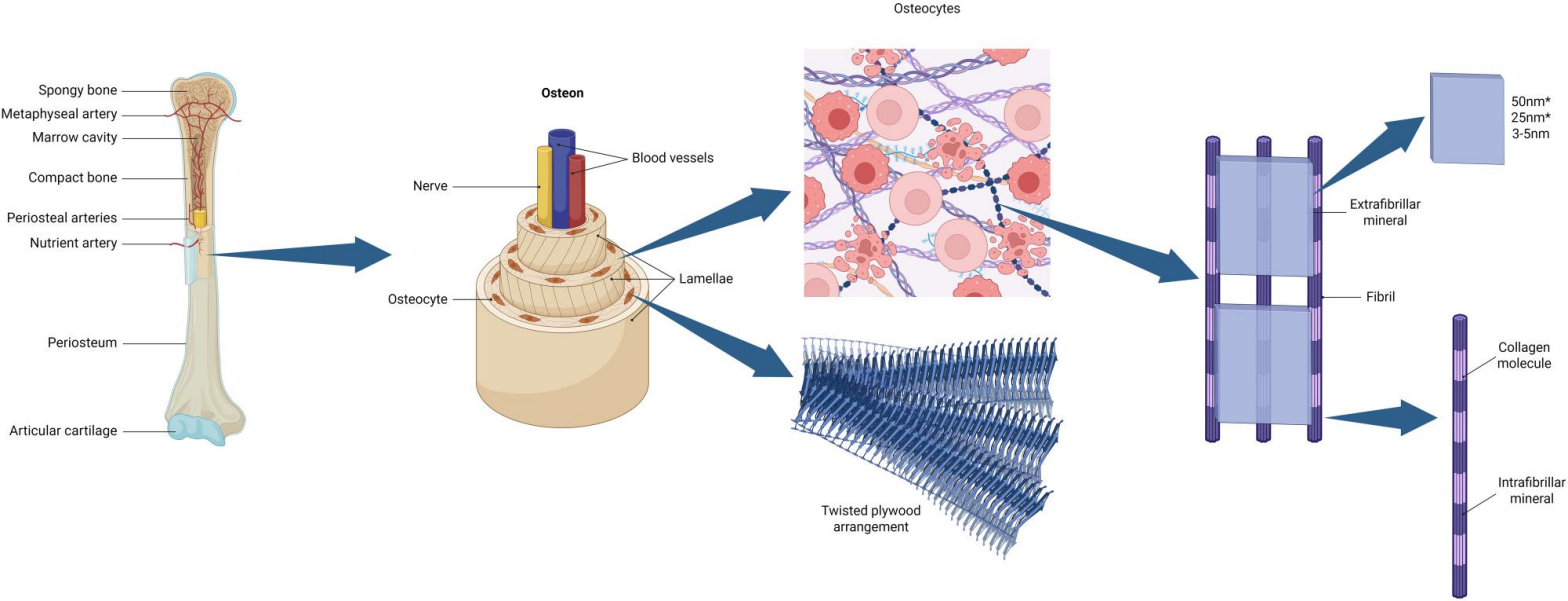
Multiscale structure of bone from macroscopic to microscopic levels and hierarchical organization of MC lagen.

Mineralized collagen (MC) refers to a type of biocomposite formed by the deposition of inorganic minerals in the collagen matrix. It is an important component of various connective tissues in the human body, for example, bones and cartilage (Figure 4) [123]. MC are composed mainly of HA and COL I in the body. The interaction between the two ensures that bones are both strong and flexible [124]. In the body, MC can naturally absorb and concentrate BMP-2 and VEGF. Owing to its structural composition and mechanical properties similar to those of natural bone, as well as its excellent biocompatibility and osteoconductivity, it has gradually become a research hotspot in the field of bone implant materials [125, 126]. Currently, many biomimetic MC products composed of HA/COL composites exist, some of which have been commercialized and used in the clinical repair of IBD [123]. These materials are typically combined with polymers to prepare scaffolds with good mechanical properties and biocompatibility, demonstrating excellent cell affinity and bone formation promotion capabilities in bone defect repair.

**Figure 5:**
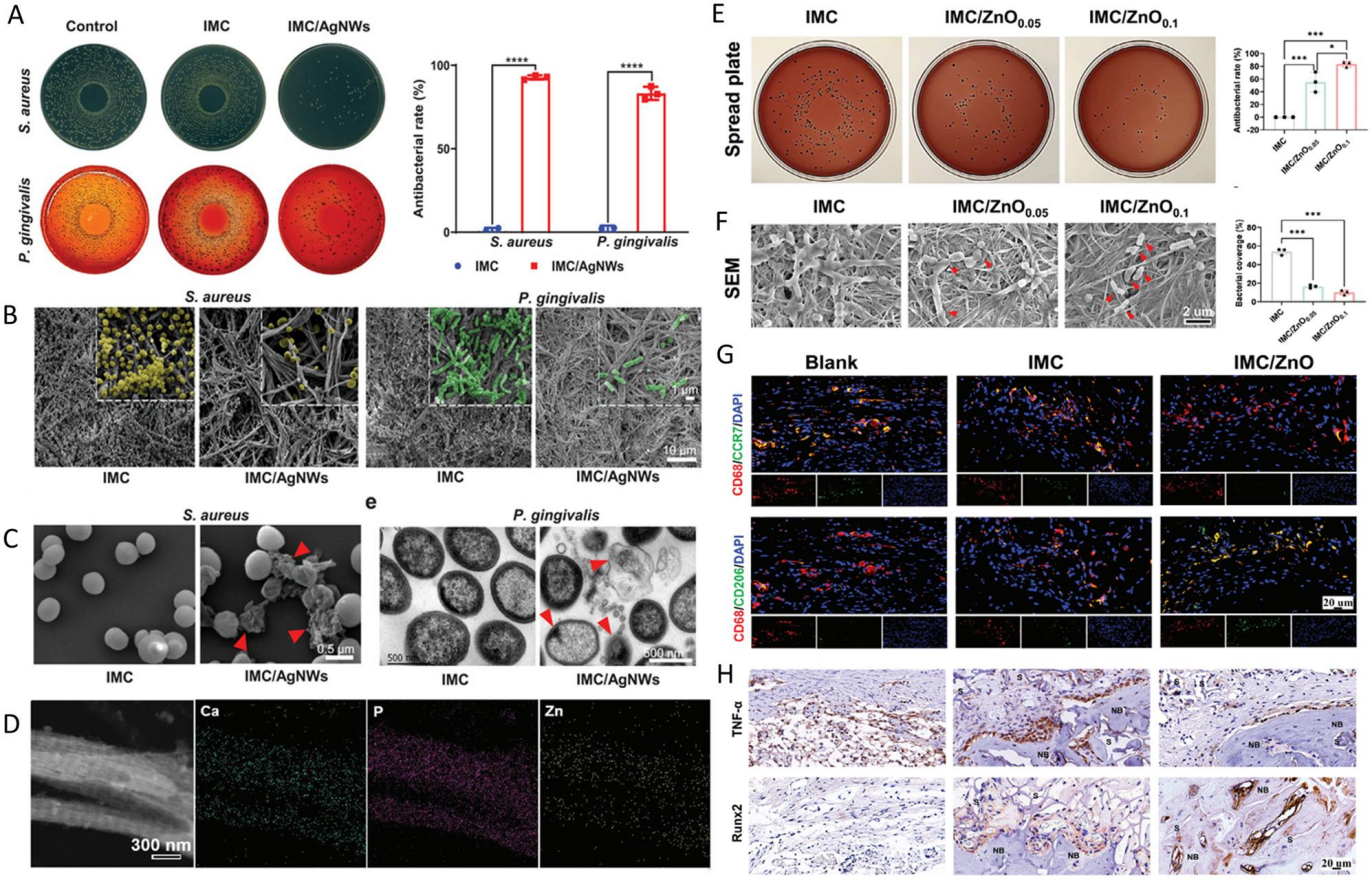
MC-based biocomposites showing combined antibacterial and osteoconductive properties. (**A**) CFU assay of supernatant of *S. aureus* (24 h) and *P. gingivalis* (48 h) cultured on IMC and IMC/AgNWs. (**B**) Representative SEM images of the micro-morphology of *S. aureus* (yellow, 24 h) and *P. gingivalis* (green, 72 h) cultured on IMC and IMC/AgNWs. (**C**) Representative SEM images of supernatant of *S. aureus* cultured with IMC and IMC/AgNWs (right) for 24 h. Reproduced with permission from Ref. [128], Copyright 2024, Wiley. (**D**) TEM and EDS images of Ca, P and Zn elements in IMC/ZnO. (**E**) Representative spread plates and antibacterial rateof *P. gingivalis* cultured on IMC and IMC/ZnO for 24 h. (**F**) SEM images and bacterial coverage of *P. gingivalis* cultured on IMC and IMC/ZnO for 24 h. Arrows: *P. gingivalis* with damaged membranes. (**G**) Representative immunofluorescent staining images of CD68, CCR7 and CD206 at week 6. (**H**) Representative immunohistochemical staining images of TNF-α (at week 6) and Runx2 (at week 10). Reproduced with permission from Ref. [129], Copyright 2024, Wiley.

The Suchý *et al*. [127] team used electrospinning technology to prepare HA/COL layers, which were cross-linked and stabilized before being impregnated with vancomycin (VAN) and gentamicin (GEN) to create a nanostructured layer with antibacterial properties. This preparation method results in a nanofiber structure with an extremely high specific surface area, which not only mimics the inorganic/organic composite structure of natural bone but also achieves synergistic controlled release of antibiotics. Research has demonstrated that the combined use of VAN and gentamicin has a synergistic effect and is not cytotoxic, suggesting a novel strategy to address the challenges of insufficient local drug concentrations and disruption of the bone regeneration microenvironment in the treatment of IBD. Zhang *et al*. [128] developed a biomimetic multifunctional scaffold (IMC/AgNWs) by embedding silver nanowires into a hierarchical fiber-inner MC matrix, innovatively combining the broad-spectrum antibacterial properties of AgNWs with the bone-inducing activity of biomimetic MC to effectively address the complex pathological environment of IBD. *In vitro* experiments have demonstrated that it exhibits significant antibacterial activity against both gram-positive and gram-negative bacteria, achieving long-term anti-infective effects by disrupting bacterial membrane structures and inhibiting biofilm formation. Additionally, the scaffold activates the BMP2/Smad/RUNX2 signaling pathway, thereby significantly promoting the osteogenic differentiation of periodontal ligament stem cells even in an inflammatory microenvironment (Figure 5A–C). In infectious rat jawbone defect and Beagle dog peri-implantitis models, IMC/AgNWs demonstrated dual therapeutic advantages: they inhibited inflammatory responses through the sustained release of ions from silver nanowires and provided mechanical support through their biomimetic mineralization structure, overcoming the limitations of traditional materials in terms of an imbalance between antibacterial and osteogenic properties and insufficient mechanical stability.

Zhang *et al*. [129] developed an MC composite scaffold (IMC/ZnO) modified with zinc oxide nanowires. This scaffold was constructed via biomimetic coassembly technology to combine ZnO nanowires with MC, forming a biocomposites with a bone-like hierarchical structure. Studies have shown that IMC/ZnO can intelligently release Zn^2+^ in an infected microenvironment, significantly inhibiting the adhesion and biofilm formation of gram-negative and gram-positive bacteria by disrupting bacterial membrane structures. Moreover, its biomimetic interface significantly promotes the adhesion, proliferation and osteogenic differentiation of BMSCs, and can upregulate the expression of osteogenic markers, such as RUNX2 and BMP2, even under inflammatory factor stimulation. *In vivo* experiments revealed that IMC/ZnO achieved near-complete bone regeneration in an infectious mandibular defect model by regulating macrophage polarization to the M2 type, reducing the secretion of the proinflammatory factor TNF-α and increasing the secretion of the anti-inflammatory factor IL-10 (Figure 5D–H). In addition, during the process of conferring antibacterial properties to mineralized rubber raw materials, many researchers have also attempted to incorporate various other materials, such as graphene oxide, AgNPs, the antibacterial peptide GL13K and naringin, to prepare numerous novel modified MC biocomposites [130]. For instance, Yu *et al*.’s [131] team constructed an MC composite coating (COL/MOF/NG) loaded with naringin using a metal-organic framework (MOF). The ultrahigh specific surface area and mesoporous structure of the Zr-based MOF nanocrystals enabled the time-controlled release of naringin. The experimental results show that this material significantly reduces the initial burst release concentration of the drug, avoiding the risk of cytotoxicity caused by high concentrations of naringin. During the early burst release phase, it provides an effective antibacterial concentration against *S. aureus*, whereas, during the sustained-release phase, it maintains a concentration that promotes bone differentiation, thereby enhancing the osteogenic gene expression of BMSCs. These materials not only demonstrate excellent biocompatibility and bone regeneration capabilities but also possess effective anti-infective properties.

Total knee arthroplasty (TKA) is a widely performed surgical intervention for managing end-stage osteoarthritis (OA), particularly among the elderly population. Early postoperative ambulation is essential for minimizing the risk of complications, including surgical site infections. However, conventional polymethylmethacrylate (PMMA) bone cement commonly used in TKA presents notable limitations, such as poor osteointegration and a higher elastic modulus compared to native bone, which hampers its suitability for early weight-bearing. To address these issues, Zhu *et al*. [132] developed a modified MC-PMMA bone cement with an elastic modulus closely approximating that of cancellous bone, thereby reducing stress shielding and microdamage at the prosthesis–bone interface. This improved mechanical compatibility mitigates the risk of microinjury-induced secondary infections and facilitates earlier mobilization following surgery. In a prospective, randomized controlled trial involving TKA patients and an average follow-up period of 45.8 months, no instances of periprosthetic infection were observed, indicating that the novel material enhances surgical safety and supports effective infection prevention through superior biomechanical integration and osteoconductive properties.

Bionic MC mimics the mineralization process of bones in nature, achieving good biocompatibility and bioactivity. Compared to HA, MC has better elasticity and toughness, making it suitable for repairing IBD in nonweight-bearing areas. Moreover, researchers are further exploring the mechanisms of natural mineralization and are continuously exploring more efficient and economical mineralization methods to better mimic the natural mineralization process.

#### BG-based biocomposites

Bioactive glass (BG) is a specialized type of glass material with a composition similar to that of natural minerals found in human bones. The discovery of BG dates back to 1969, when Professor Hench and his team, while developing a material to aid wound healing and compatible with bone tissue, first discovered a special glass composed of sodium, calcium, phosphorus and silicon, which was proven to bond tightly with bone, laying the foundation for research into BG [133]. The first BG developed by the Hench team is called 45S5 BG, which is composed of 45 wt% SiO_2_, 24.5 wt% CaO, 24.5 wt% Na_2_O and 6.0 wt% P_2_O_5_. It can bond with human bone and promote osteogenesis by releasing active factors. Millions of patients worldwide have successfully received implants based on 45S5 BG, which are primarily used to repair bone and dental defects [134]. The ionic dissolution products of BG can stimulate the proliferation and differentiation of osteoblasts, enhancing the expression of osteogenic genes, and thus, promoting bone regeneration at the genetic level [135]. Compared with traditional autografts or allografts, BG offer unique antibacterial and anti-inflammatory advantages, which are important for treating IBDs.

Recent studies have confirmed that BG possesses strong antibacterial and antibiofilm properties and has a certain inhibitory effect on the biofilm formation of various microorganisms that cause prosthetic infections [136, 137]. Research into the antibacterial mechanism of BG has long been a topic of interest. Extensive research has shown that this antibacterial effect is the result of multiple mechanisms that act together. Researchers have proposed various theories about how they work, for example, changes in local pH and osmotic pressure, as well as sharp glass fragments that may damage the bacterial cell wall. These fragments can create holes in the cell wall, promoting the penetration of antimicrobial agents into the cytoplasm of microorganisms [138]. Studies on the dissolution behavior and antibacterial effects of BG have confirmed that the antibacterial effects of BG are closely related to its dissolution properties, particularly increases in the pH and alkaline ion concentration. Notably, when the pH of the medium is neutralized, the antibacterial activity of the BG tends to decrease, suggesting that this may be a fundamental mechanism underlying its antibacterial action [139]. In addition, some studies have shown that adding additional antimicrobial agents to BG can increase biofilm resistance [137]. Mesoporous bioactive glass (MBG) has advantages over traditional BG in terms of their high specific surface area and large pore size, enabling efficient loading of antibiotics, growth factors or metal ions and achieving controlled drug release [140]. Heras *et al*. [141] designed a zinc-doped mesoporous BG composite scaffold (ZnO-MBG) that achieves synergistic antibacterial and osteogenic effects through multiscale material engineering. The scaffold is based on 80% SiO_2_–15% CaO-5% P_2_O_5_ mesoporous glass, with 4% ZnO (4ZN-GE) introduced and antibiotics such as levofloxacin (LEVO) and VAN loaded onto it. Zn^2+^ exert broad-spectrum antibacterial effects by disrupting bacterial membrane integrity, inhibiting DNA replication and generating ROS. They also exhibit synergistic effects with antibiotics, significantly reducing the minimum inhibitory concentration (MIC) for inhibiting *S. aureus* and *E. coli* planktonic bacteria. *In vitro* experiments have shown that 4ZN-GE scaffolds loaded with VAN or LEVO can completely destroy *S. aureus* biofilms, whereas those loaded with GENTA achieve a 100% clearance rate for *E. coli* biofilms. The sustained release of Zn^2+^ not only enhances antimicrobial persistence but also promotes bone regeneration by activating osteogenesis-related genes. Combining the dual antimicrobial and osteogenic functions of zinc, this approach offers a new low-dose, high-efficiency treatment strategy for antibiotic-resistant bacterial infections in bone defects.

**Figure 6:**
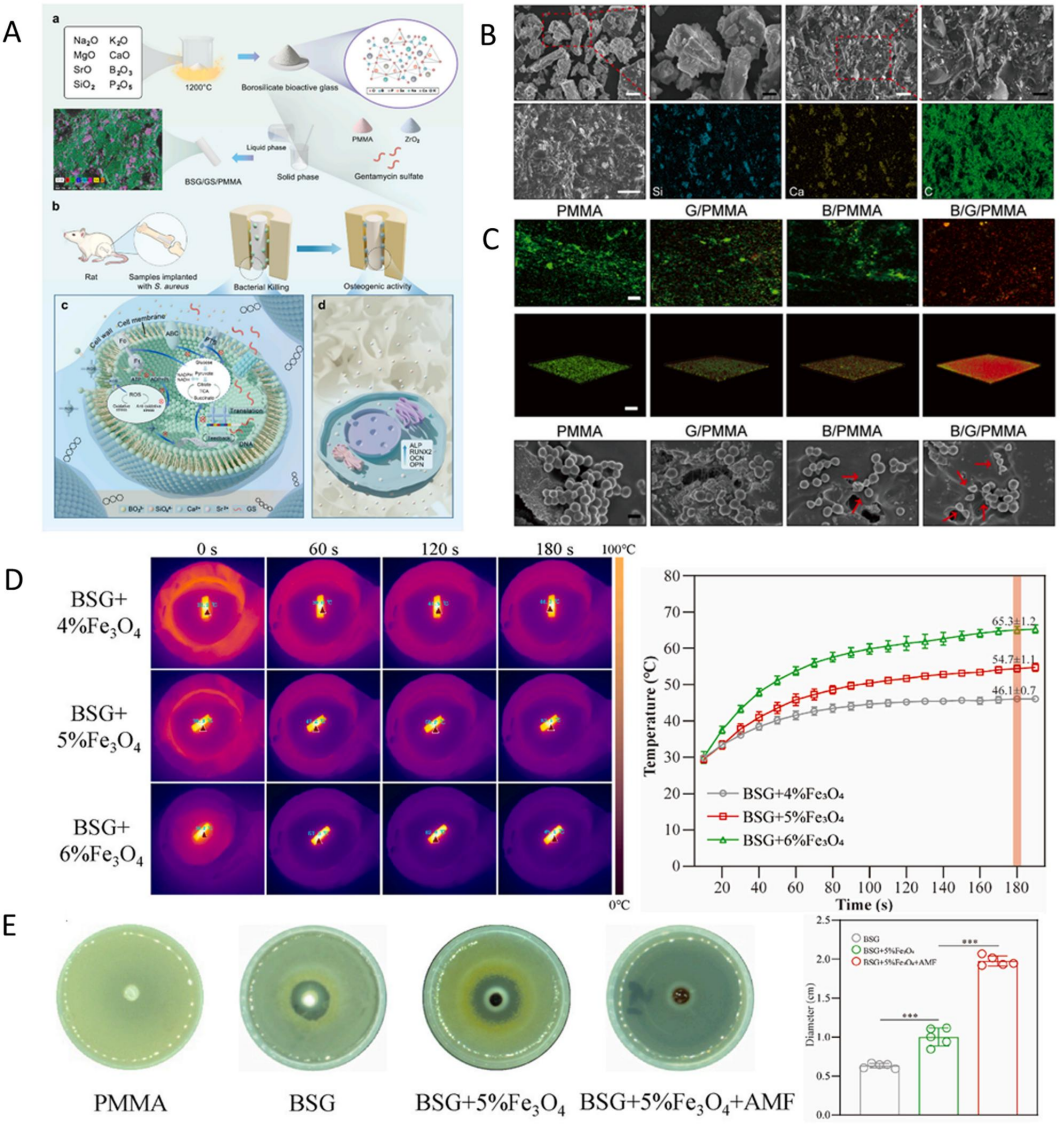
Synthesis, applications and antibacterial mechanisms of multifunctional BG-based biocomposites. (**A**) Preparation, application and antibacterial mechanism of B/G/PMMA. (a) Fabrication of borosilicate bioactive glass and bioactive cement. (b) After implanting the cement into the infected femoral bone marrow cavity, there is a temporal transition from combating infection to promoting bone formation. (c) The antibacterial mechanism of bioactive antibacterial PMMA cement. (d) The osteogenic effect of bioactive antibacterial PMMA cement. (**B**) Preparation and characterization of B/G/PMMA. (**C**) Live/Dead staining and morphology of *S. aureus* following 6 h of culture on the surface of cement samples. Reproduced with permission from Ref. [144], Copyright 2024, Elsevier. (**D**) Magnetic-thermal-induced thermal efficiency of BSG + Fe_3_O_4_  *in vitro*. (**E**) Antibacterial properties of each scaffold *in vitro* and statistical analysis of diameter of bacteriostatic zone. Reproduced with permission from Ref. [146], Copyright 2024, Elsevier.

Recent research has focused on optimizing the microstructure of BG by regulating the ratio and distribution of crystalline and glass phases within the material, thereby enhancing its mechanical properties and bioactivity. This optimization is crucial for ensuring the stability of the material in the treatment of bone defects under heavy loads. Research reports that by introducing boron to regulate the phase separation behavior of silicate glass, a nanoscale glass phase-crystal phase composite structure can be formed. The addition of boron causes the silicon-oxygen network to break, forming boron-rich regions (glass phase) and silicon-rich regions (crystalline phase), with the crystalline phase consisting mainly of diopside (CaMgSi_2_O_6_) [142]. This structure increases the compressive strength of the material to 120–150 MPa while enhancing antibacterial activity through the release of ${\text{BO}}_{3}^{3–}$ [143]. Borosilicate bioactive glass (BSG) has become the focus of tissue engineering research due to its excellent performance in soft tissue and hard tissue repair. Fan *et al*. [144] innovatively constructed a BSG-synergistic low-dose GS PMMA composite bone cement (B/G/PMMA). This study designed a borosilicate glass network by regulating the B_2_O_3_/SiO_2_ ratio. Its amorphous structure was verified by X-ray diffraction (XRD), and FTIR analysis revealed the characteristic vibration peaks of B-O-B and Si-O-Si. The glass was uniformly distributed in the PMMA matrix. The material has a compressive strength of 75.4 ± 0.9 MPa, meeting the ISO 5833 standard for orthopedic implants. It also creates an alkaline microenvironment by continuously releasing ${\text{BO}}_{3}^{3–}$, Ca^2+^ and other ions, destroying the integrity of bacterial cell membranes, inhibiting ATP synthesis and weakening the antioxidant system, thereby significantly enhancing the efficiency of low-dose GS in removing *S. aureus* biofilms (Figure 6A–C). Moreover, the synergistic release of GS from BSG degradation products activates the RUNX2/ALP/OCN osteogenic pathway in hBMSCs, promoting *in vitro* mineralization and *in vivo* bone regeneration. This design overcomes the limitations of high-dose antibiotic bone cement use. Micro-CT analysis revealed that the bone volume fraction (BV/TV) in the B/G/PMMA group was significantly greater than that in the pure PMMA group and that a dense calcified layer formed around the implant.

Iron oxide nanoparticles (IONPs) are superparamagnetic nanoparticles composed of Fe_3_O_4_ or γ-Fe_2_O_3_. This process is triggered by an alternating magnetic field, which induces high temperatures to precisely eliminate deep-seated bacterial biofilms, overcome antibiotic penetration barriers and achieve magnetic-thermal targeted antibacterial effects. They also possess multiple functions, including magnetic-targeted delivery, biodegradability and activity that promotes bone metabolism. They have been approved by the Food and Drug Administration (FDA) and have become the most extensively studied material in the field of nanomedicine [145]. Thus, Jin *et al*. [146] designed a composite scaffold combining BSG with Fe_3_O_4_ magnetic nanoparticles (BSG + 5%Fe_3_O_4_), addressing the challenge of balancing biofilm removal and bone regeneration microenvironment coordination in the treatment of IBD. In this scaffold, Fe_3_O_4_ generates a targeted magnetothermal effect of 54.7 ± 1.1°C under an alternating magnetic field, penetrating the trabecular bone network to kill hidden MRSA (Figure 6D and E). Moreover, the alkaline microenvironment formed by BSG degradation achieves synergistic antibacterial effects by disrupting bacterial membrane integrity, significantly improving antibacterial efficiency compared with traditional antibiotic bone cement. Moreover, the boron and silicon ions released by BSG activate the NOD/TNF signaling pathway to induce M2 polarization of macrophages, suppress proinflammatory factors such as IL-6 and IL-1β, and significantly upregulate the expression of the osteogenic genes RUNX2 and OCN in MSCs, achieving a dynamic coupling of ‘anti-inflammatory-osteogenic’ effects. This material regulates the B/Si ratio to synchronize degradation rates with bone formation, achieving gradient degradation within 42 days. Its initial compressive strength aligns with the mechanical requirements of cancellous bone. Micro-CT imaging revealed a significant negative correlation between new bone volume and degradation area, overcoming the limitations of traditional material mechanical mismatch. This provides an innovative treatment paradigm for IBD, integrating ‘osteogenic-anti-infective-degradable’ functions.

To address the multifaceted challenges presented by comorbid conditions such as diabetes, Zhu *et al*.’s [132] team developed a bioactive glass-based nanozyme composite cryogel (MnO_2_@PDA-BGs/Gel) with a macroporous architecture, designed to modulate the hostile microenvironment of diabetic infected wounds. In this system, polydopamine (PDA) facilitates the deposition of MnO_2_ onto the surface of BG, endowing the composite with multifunctional enzyme-mimetic properties. Within the acidic and infection-prone milieu of diabetic wounds, the material catalyzes endogenous hydrogen peroxide to generate bactericidal reactive oxygen species (ROS). Simultaneously, its SOD- and CAT-like activities mitigate oxidative damage by eliminating excess ROS, thereby alleviating oxidative stress exacerbated by hyperglycemia. PDA further imparts near-infrared photothermal responsiveness, enabling localized hyperthermia upon irradiation, which enhances antibacterial performance. Montalbano *et al*. [147] advanced a composite scaffold by incorporating strontium-containing MBGs into a type I collagen matrix, further stabilized through an ethanol-mediated genipin crosslinking strategy. This crosslinking approach not only prevents premature leaching of Sr^2+^ during scaffold fabrication but also ensures the sustained release of osteoinductive factors postimplantation, avoiding the loss of bioactivity. Genipin-induced covalent bonding significantly enhances the mechanical integrity and degradation resistance of the scaffold, reducing the risk of structural collapse in physiological environments. Moreover, nanoMBGs promote the formation of a dense HA mineralized layer on collagen fibrils, closely mimicking the microtopography and nanotopography of natural bone. This biomimetic architecture reduces nonphysiological porosity and minimizes bacterial adhesion sites, thereby lowering the risk of biofilm formation. Niu [148] reported the synthesis of a molybdenum-doped nano bioactive glass (BBGN-Mo), which features oxygen vacancies and free electrons derived from the mixed-valence state of Mo^4+^/Mo^6+^. These properties allow the material to produce a controlled, mild photothermal effect (∼42°C) under single-wavelength near-infrared irradiation, enabling the targeted eradication of drug-resistant bacteria and residual tumor cells, thus, minimizing secondary infection risk following incomplete debridement. The sustained release of antioxidant constituents suppresses postoperative pro-inflammatory cytokine expression, mitigates hematoma and exudate accumulation and promotes neovascularization. These effects collectively improve local perfusion and alleviate ischemia at the implant site, supporting tissue regeneration and functional recovery.

The above studies demonstrated the potential of biocomposite glass for promoting bone regeneration, angiogenesis and antibacterial activity. Despite these advantages, there are some limitations to their application in future clinical practice. The main issue is that the mechanical strength of the current biocomposite glass may not be sufficient for use in weight-bearing areas. Currently, scholars are researching methods to improve the mechanical properties of these materials without affecting their biological activity or biodegradability [149]. The excellent bioactivity of BG results from the release of ions such as Si, Ca and phosphate, which can stimulate bone cell activity and accelerate growth. However, the release of ions and other degradation products must also be carefully monitored to prevent local or systemic toxicity.

### Metal-based biocomposites

The traditional biomaterial metals currently used in medicine can be divided into two main categories: biocompatible and biodegradable. Pure titanium and its alloys, stainless steel and cobalt-based alloys are typically classified as biocompatible metals. In contrast, magnesium, iron and zinc are considered biodegradable metals [150]. Although metallic materials offer multiple advantages as bone implants, they also have certain limitations [151]. The elastic modulus of cortical bone typically ranges from 17 to 20 GPa, which is significantly lower than most metals used for bone implants. This difference in elastic modulus can sometimes lead to the ‘stress shielding’ phenomenon, where the bone surrounding the implant gradually thins or weakens due to reduced force, resulting in implant loosening or refracture after implant removal [152]. To address this issue, researchers have designed a new type of porous metal by altering the geometric shape of the implant. This porous structure effectively reduces the elastic modulus of the metal and by adjusting the pore structure, the implant’s mechanical properties can be consistent with those of human bone tissue [153]. In addition, the porous structure can also promote the growth of new bone tissue into the implant, improving the transport of nutrients and metabolic waste, thereby improving the interaction between the bone and the implant and increasing the life and durability of the implant [38, 154, 155]. Traditional methods for manufacturing porous metal-bone implant materials include powder metallurgy, freeze drying, gas foaming, discharge plasma sintering and open-cell titanium foam [156]. However, these methods have disadvantages such as uneven pore sizes, poor permeability and difficulty in preparing structurally complex porous materials [157]. The use of additive manufacturing (AM) technology to prepare materials with controllable porosity and structure has attracted widespread attention from researchers and 3D-printed metal-bone implants have gradually become among the most widely used materials in clinical applications [11]. However, certain properties of bone implants, for example, wear resistance, hardness, magnetic resistance or antibacterial properties, are difficult-to-add through geometric design alone. These properties require the base material’s modifications before AM processing [158].

#### Stainless steel-based biocomposites

Stainless steel (SS) is an iron-based alloy containing high levels of chromium (11–30 wt%) and varying levels of nickel [159]. Compared to other metallic materials, it is the most cost-effective material choice for orthopedic implants due to its low cost, ease of manufacture and suitable corrosion resistance [160]. Although stainless steel is widely used as a biomaterial and has good biocompatibility, it does not possess inherent biological functional properties such as blood compatibility, osteoconductivity and bioactivity [161]. Therefore, researchers have modified the surface of stainless steel by adding coatings or performing chemical treatments, introducing the desired properties without sacrificing important overall characteristics [162].

**Figure 7:**
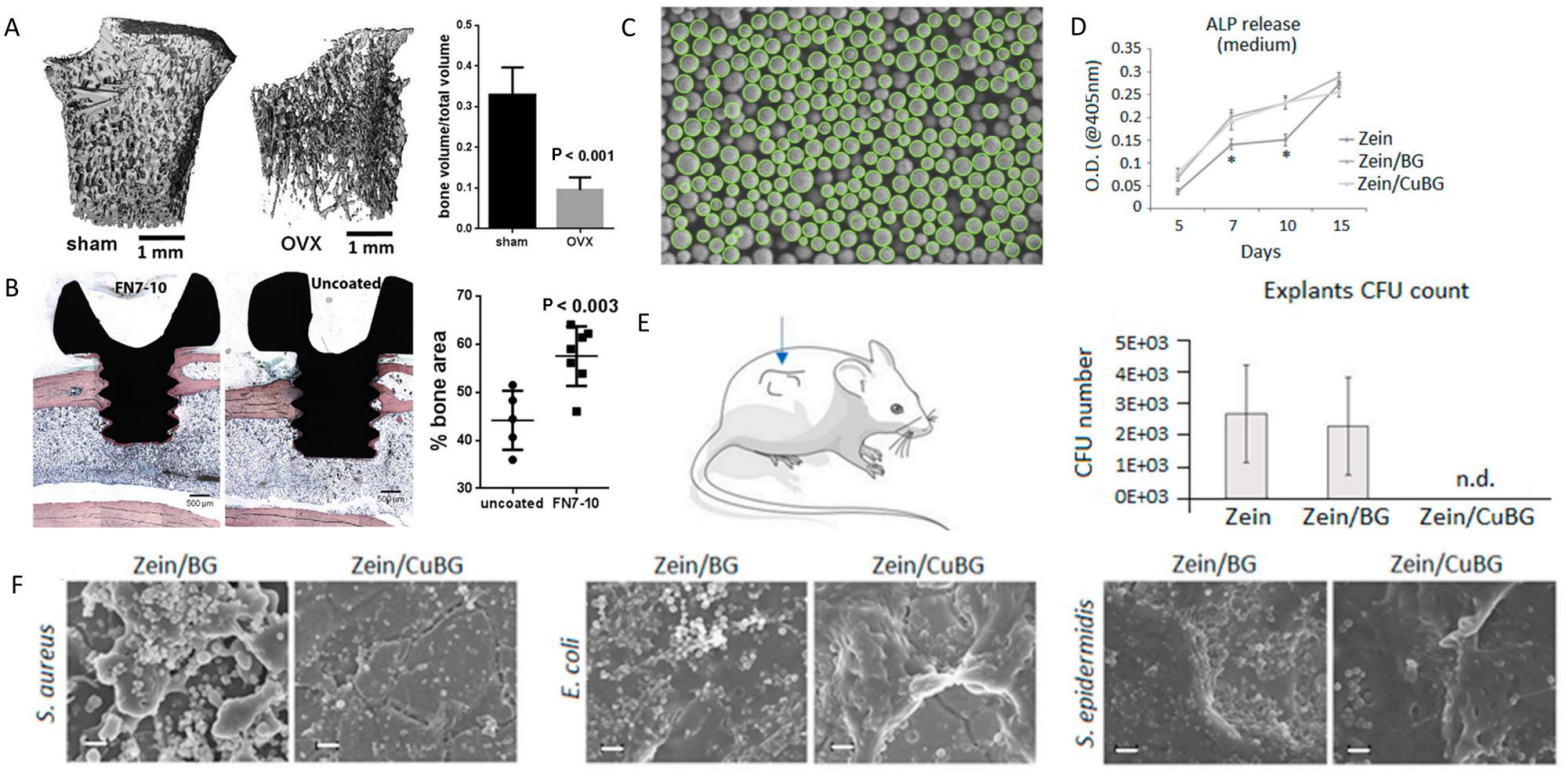
Analysis of multifunctional stainless steel-based biocomposites. (**A**) validation of osteoporosis in ovariectomised rats. (**B**) Bone-implant contact and bone tissue growth into the screw threads. Reproduced with permission from Ref. [166], Copyright 2015, Elsevier. (**C**) SEM image of the analyzed particles with the applied proposed image processing method. Reproduced with permission from Ref. [169], Copyright 2020, Elsevier. (**D**) Osteogenic results. (**E**) In the subcutaneous implant model, copper addition (zein/CuBG) caused the inhibition of the induced infection in comparison with zein (control) and zein/BG. (**F**) SEM images showing the lack of evident biofilm aggregates on zein/CuBG coatings. Reproduced with permission from Ref. [93], Copyright 2021, Elsevier.

Preparation of bioactive HA coatings on SS surfaces is an effective strategy for enhancing their corrosion resistance and bioactivity. Over the past 20 years, several bioactive HA-derived coatings have been proposed to improve the performance of SS [163, 164]. However, only a few attempts have been reported in the literature for complex and corrosion-prone surfaces, for example, screws. To enhance the integration of implants with bones, researchers have proposed the presence of adhesion motifs derived from extracellular matrix proteins on the implant surface, which can bind to integrin adhesion receptors [165]. Agarwal *et al*. [166] developed a 316SS screw coating based on a human fibronectin fragment (FN7–10). This coating significantly promotes the adhesion and osteogenic differentiation of MSCs by specifically activating the α5β1 integrin signaling pathway. In healthy and osteoporotic rats, the coated screws significantly improved mechanical fixation and bone ingrowth (Figure 7A and B). This method can be adapted to complex implant geometries without complicated pretreatment, providing an efficient solution for osseointegration in cases of IBD with low bone volume. Electrophoretic deposition (EPD) is a highly efficient and adaptable surface engineering technology that uses an electric field to drive the directed migration and deposition of charged particles in a colloidal suspension. This enables the preparation of functionalized coatings on a variety of substrates at room temperature [167]. Research shows that EPD is compatible with bioactive materials such as gelatin and chitosan, providing a controllable and scalable preparation platform for the design of functional biocoatings [168]. Aydemir *et al*. [169] constructed a chitosan/gelatin/SiGe NP coating on the surface of 316L SS using EPD technology. The coating achieves a uniform distribution of nanoparticles and controlled drug release through optimization of the biopolymer ratio and deposition parameters. *In vitro* experiments revealed that the coating had a significant inhibitory effect on *S. aureus* and *E. coli*, and the release concentration was significantly greater than the MIC. Moreover, the coating induced hydroxycarbonate apatite deposition in simulated body fluids and supported the proliferation of ST-2 (Figure 7C).

The formation of biofilms on the surface of metal materials used to treat IBD remains a serious problem. Corn zein is a natural alcohol-soluble protein extracted from the corn endosperm. It is a hydrophobic biopolymer with a molecular weight of about 25 000–45 000. It is rich in hydrophobic amino acids, for example, leucine and proline and possesses unique film-forming properties, biodegradability and biocompatibility [170]. Research shows that Zein can be used to construct sustained-release scaffolds by loading antimicrobial agents, combining its resistance to microbial enzymes to achieve a synergistic antimicrobial-osteogenic effect. Moreover, its membrane-forming ability can be combined with bioceramics to optimize bone conductivity, compensating for its own inability to promote cell proliferation [171]. Rivera *et al*. [93] developed a composite coating based on Zein and copper-doped BG (CuBG), which was successfully attached to the surface of 316L SS implants using EPD technology. The sustained release of copper ions significantly inhibited biofilm formation by *S. aureus*, *E. coli* and *S. epidermidis* and completely eliminated *S. aureus* colonization of the implants in an *in vivo* model. BG components significantly promote the differentiation of hFOB osteogenic precursor cells by inducing HA deposition and upregulating the expression of osteogenic genes, such as COL1 and OPN, resulting in a significant increase in ALP activity within 7–10 days. In addition, copper ions induce neovascularization at the subcutaneous implantation site in mice by activating the HIF-1α/VEGF pathway. The coating exhibited excellent mechanical adhesion and controlled degradation properties while maintaining high biocompatibility with fibroblasts, endothelial cells and osteoblasts (Figure 7D–F).

Fixing enzymes to its surface is another method of imparting antibacterial activity to SS. Lysozyme is a natural enzyme that plays a vital role in preventing bacterial infections in the human body. This enzyme is harmless to the environment and is more specific than traditional biocides, such as antibiotics and quaternary ammonium compounds. Yuan *et al*. [172] utilized surface-initiated atom transfer radical polymerization (ATRP) technology, poly(ethylene glycol) methacrylate (PEGMA) brushes were grafted onto the SS surface and lysozyme was immobilized at the ends of the PEGMA chains using 1,1'-carbonyldiimidazole (CDI). This functionalized SS surface effectively prevented protein adsorption, reduced bacterial adhesion and biofilm formation and exhibited good bactericidal effects against *S. aureus* and *E. coli*.

Another significant challenge in clinical applications of stainless steel (SS)-based composites is the inflammatory response induced by surface microstructural imperfections. To address this, Ghosh *et al*. [95] and colleagues applied surface mechanical attrition treatment (SMAT) to 316L stainless steel, resulting in the formation of a nanocrystalline surface layer characterized by a high density of defects. This modification markedly increased surface hardness and minimized debris generation during fretting wear. Additionally, it facilitated the formation of a thicker and more stable chromium-enriched passive film, enhancing electrochemical stability and interfacial bioactivity. *In vivo* studies using a murine calvarial defect model demonstrated that the treated material did not elicit an immune response, effectively mitigating inflammation risks associated with metal ion release and particulate generation.

#### Cobalt-based biocomposites

Compared to traditional 316L stainless steel, cobalt-based alloys exhibit higher specific strength, more stable passive film formation and greater corrosion resistance. Castable CoCrMo alloys are widely used in orthopedic joint prostheses because of their excellent corrosion and wear resistance [173]. Moreover, forged CoNiCrMo alloys, which incorporate nickel (Ni), further enhance corrosion resistance while maintaining high mechanical strength and toughness. In recent years, the diversification of research by various scholars has provided unlimited possibilities for the application of cobalt-based alloys in the treatment of IBD. Many researchers have attempted to develop antibacterial biomaterials through surface modification or element alloying processes.

**Figure 8:**
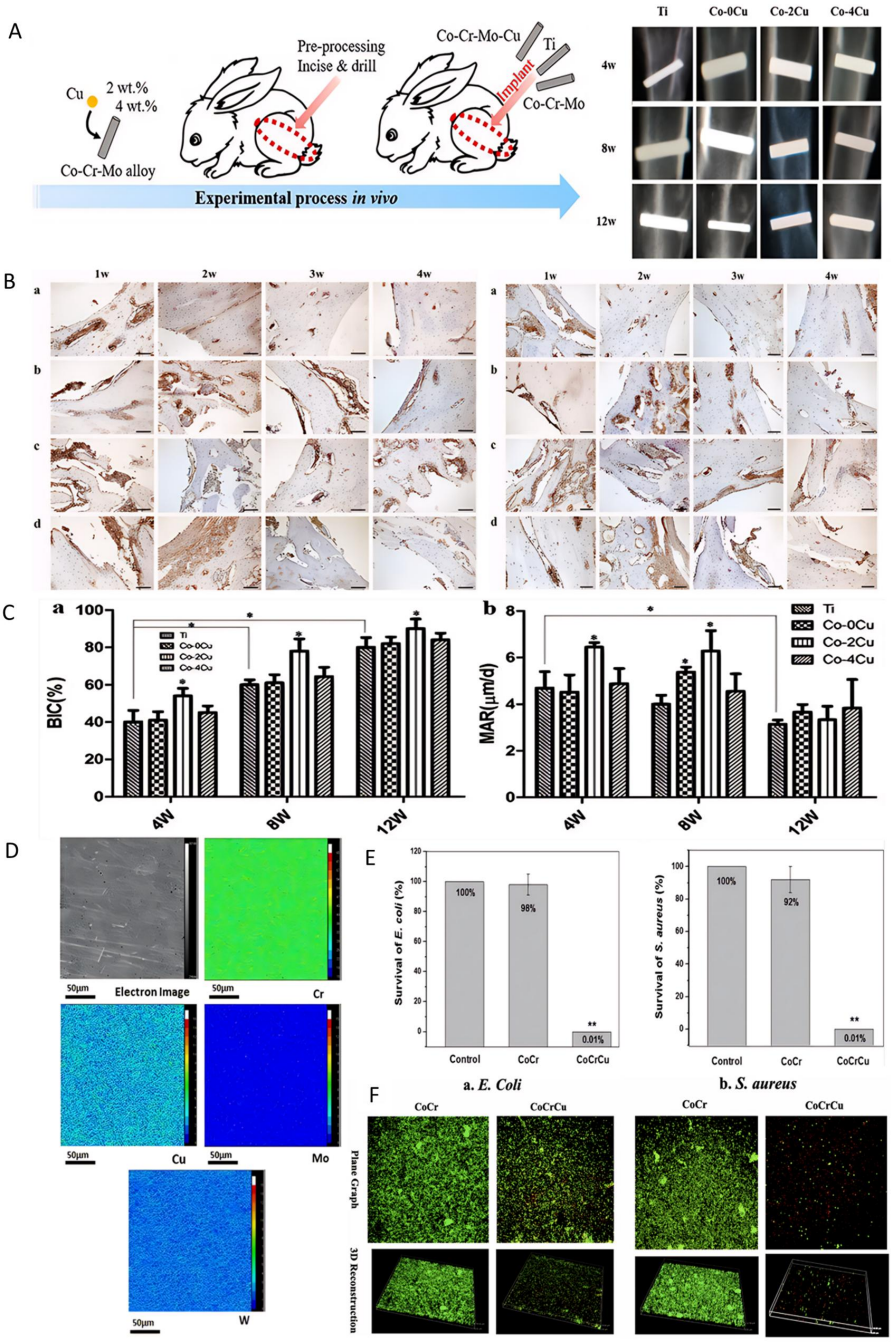
*In vivo* animal studies, material characterization and antimicrobial performance of cobalt-based alloys. (**A**) Schematic illustration of the experimental procedure for implanting Co-Cr-Mo-Cu alloys with different Cu contents into rabbit tibiae, along with the radiographic examination results. (**B**) BMP-2 staining (left) and IGF-1 staining (right) of tibiae implanted with Ti alloy (a), Co-0Cu alloy (b), Co-2Cu alloy (c) and Co-4Cu alloy (d) (scale bar: 20 μm). (**C**) Bone-implant contact (BIC) measurements (a) and mineral apposition rate (MAR) measurements (b) of Co-Cr-Mo-Cu alloys with different Cu contents at 4, 8 and 12 weeks. Reproduced with permission from Ref. [176], Copyright 2020, Oxford. (**D**) EDS images show the uniform distribution of Cu and other elements in the alloy. (**E**) Bacterial viability of *E. coli* (left) and *S. aureus* (right) after direct contact with different materials (control, CoCr and CoCrCu) at 37°C for 24 h, which was used to assess antibacterial performance. (**F**) CLSM images (including planar and 3D reconstructed views) of *E. coli* (left) and *S. aureus* (right) after direct contact with SLM CoCr and CoCrCu alloys at 37°C for 24 h were used to evaluate antibiofilm properties. Reproduced with permission from Ref. [180], Copyright 2016, Elsevier.

As cobalt-based metals are increasingly used in clinical applications, various methods have emerged to impart antimicrobial properties to these materials. For example, Hu *et al*.’s [174] research has demonstrated that cobalt-chromium alloys treated with alkali and heat can effectively reduce bacterial adhesion, thereby exerting an antimicrobial effect, without exhibiting cytotoxicity toward osteoblasts. Totea *et al*. [175] prepared copper- and zinc-ion-doped HA coatings on the surface of a CoCrMo alloy. The doping of these ions significantly enhanced the antibacterial properties of the coatings, with the copper-doped FHA coating exhibiting the best antibacterial activity. Therefore, current research on antimicrobial cobalt-based alloys has focused mainly on the preparation of copper-containing cobalt-based alloys. However, surface-modified antibacterial coatings have a significant drawback: the coating may wear off or peel away, rendering it ineffective within a short period of time. Therefore, researchers have distributed the antibacterial agent evenly throughout the alloy to ensure a more lasting antibacterial effect. Duan [176] reported that adding different amounts of copper can also improve the osteogenic properties and antibacterial properties of the alloy, especially the Co29Cr-6Mo-Cu alloy containing 2 wt% copper, which exhibited the best osteogenic ability among all the samples. It is speculated that this is due to the release of Cu2+ from the copper-rich phase, which promotes the expression of BMP2 and IGF-1 (Figure 8A–C). They also reported that an increase in copper content leads to a decrease in the alloy’s wear resistance, but this can be significantly improved through appropriate heat treatment [177].

In the biomedical field, the manufacture of cobalt-chromium-based alloys mainly relies on mold forming and milling processes. However, components manufactured via these traditional methods often require additional processing, primarily due to insufficient dimensional accuracy and shape precision [178]. In addition, CoCrMo alloys have a low degree of automation, and it is challenging to manufacture complex orthopedic components via conventional manufacturing methods [179]. Therefore, as a 3D printing method that directly manufactures metal products from CAD data, selective laser melting (SLM) technology can tailor personalized implant materials for patients and has been widely promoted in clinical practice. Ren *et al*. [180] used SLM technology to prepare a new type of multifunctional CoCrCu alloy from a mixture of commercial CoCr-based alloy and elemental copper powder. Experiments have shown that it has better antibacterial and antibiofilm properties than surface-modified alloys. Furthermore, the use of SLM technology not only improved the efficiency of preparation but also enabled more precise control of the alloy composition, thereby improving the product performance (Figure 8D–F).

For diabetic wound applications, Mandakhbayar *et al*. [181] proposed a novel cobalt-doped nanoglass-based bioactive nanozyme (CoNZ). The *in situ* formation of Co_3_O_4_ nanocrystals on its surface imparts potent ROS-scavenging activity, significantly mitigating oxidative stress in diabetic wounds. This, in turn, downregulates key inflammatory signaling pathways, including NF-κB activation in macrophages, thereby facilitating the transition from inflammation to tissue repair. Concurrently, the sustained release of cobalt ions stabilizes hypoxia-inducible factor HIF-1α and upregulates the expression of vascular endothelial growth factor (VEGF), promoting endothelial cell migration and neovascularization. These mechanisms collectively ameliorate the ischemic and hypoxic microenvironment characteristic of diabetic wounds. Finally, Man *et al*. [182] compared the *in vivo* studies demonstrated that CoNZ achieved wound healing outcomes comparable to those of the clinical agent deferoxamine, underscoring its potential as a drug-free therapeutic alternative. In a separate study, Man utilized a dual-fabrication strategy combining laser-directed energy deposition with electrochemical etching to engineer nanoscale groove patterns with controlled aspect ratios on SS surfaces. These topographical modifications significantly enhanced osteoblast adhesion and differentiation while modulating platelet behavior through physical surface cues. The resulting reduction in platelet adhesion and activation lowered the risk of thrombosis and improved overall hemocompatibility. Additionally, the engineered nanotopography limited bacterial surface interaction and interfered with initial biofilm formation, thereby imparting robust antibiofilm properties without the use of exogenous antimicrobial agents. This advancement offers a promising approach for improving postoperative outcomes and minimizing complications.

Although cobalt-based alloys demonstrate excellent comprehensive performance in medical applications, during long-term clinical use involving contact with tissue fluid, cobalt and chromium ions in the metal material may leach out to a certain extent. The long-term accumulation of these ions may damage the activity of osteoblasts and osteoclasts and their mineralization process. In addition, chromium and cobalt ions are nephrotoxic, and high concentrations in the blood can cause irreversible renal failure and even type IV hypersensitivity reactions. However, this is a very rare side effect of metal implants in the body [183, 184].

#### Titanium-based biocomposites

Titanium and its alloys, as third-generation biomaterials, are widely used in bone grafting because of their excellent corrosion resistance, mechanical properties and biocompatibility [185]. Unlike stainless steel and cobalt-based alloys, titanium alloys exhibit minimal corrosion *in vivo*, with low ion release, making them suitable for long-term implantation [186]. The biocompatibility of titanium alloys arises from the formation of a dense titanium dioxide film on their surface, which enhances their stability during implantation [187]. Moreover, low-modulus β-type titanium-based alloys (such as Ti-Nb-Ta-Zr alloys) have become a focus of research because of their low elastic modulus, which alleviates the stress shielding effect, and their lack of harmful elements [188]. Although traditional titanium and its alloys have demonstrated successful osseointegration in most clinical cases, as biologically inert materials, they lack effective bone induction and antibacterial properties in complex IBD environments. Therefore, research on titanium-based biocomposites in the 21st century has shifted its focus to combining them with bioactive materials and antibacterial agents to impart multifunctionality.

**Figure 9:**
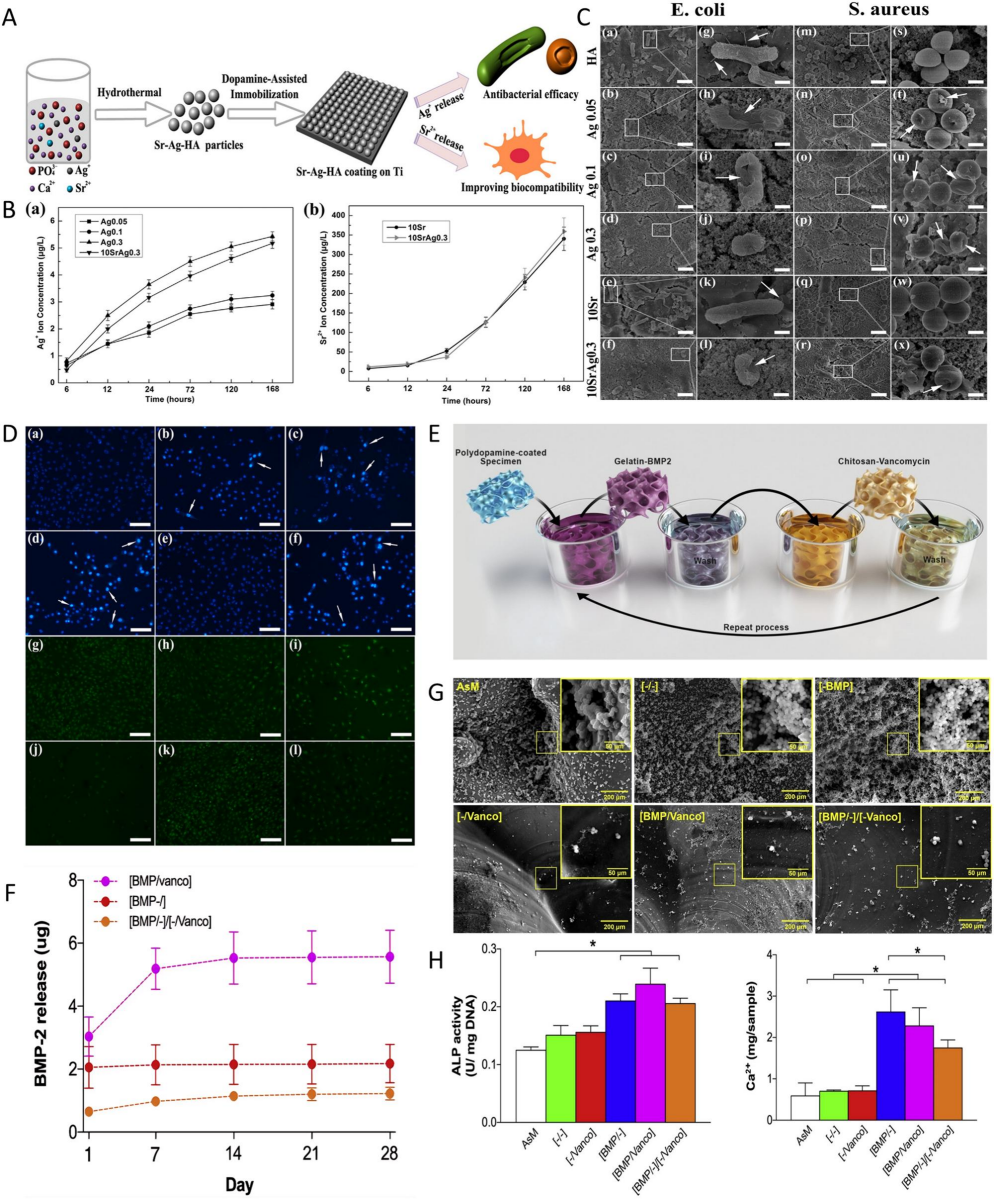
Analysis of titanium-based functional coatings. (**A**) Schematic diagram of the hydrothermal process and immobilization of Sr–Ag–HA coating on a titanium substrate. (**B**) Ion release curves of Ag^+^ and Sr^2+^. (**C**) SEM images of *E. coli* and *S. aureus* incubated for 24 h on each sample. Scale bars: (a–f) and (m–r), 2 μm; (g–l) and (s–x), 400 nm. (**D**) Representative fluorescence microscopy images of the MG63 cells cultured for 3 (a–f) and 7 (g–l) days. (a and g) HA, (b and h) Ag0.05, (c and i) Ag0.1, (d and j) Ag0.3, (e and k) 10Sr and (f and l) 10SrAg0.3. Scale bars, 200 μm. Reproduced with permission from Ref. [192], Copyright 2016, Elsevier. (**E**) Schematic diagram of the layer-by-layer coating process. (**F**) Cumulative release curves of BMP-2 for different coating materials. (**G**) Effects of different experimental groups on planktonic and attached bacterial counts. (**H**) ALP activity after 10 days (left) and calcium deposition after 14 days (right) for different experimental groups. Reproduced with permission from Ref. [196], Copyright 2020, Elsevier.

Currently, surface modification (such as coating, roughening or chemical treatment) is the primary strategy for achieving this goal. Some inorganic antibacterial agents, such as metal ions or nanoparticles of silver, copper, zinc and cerium, have been widely added to implant coatings [189]. Amin Yavari *et al*. [190] used SLM technology to manufacture porous titanium scaffolds. Then, nanotubes formed on their surfaces through anodization. These titanium scaffolds with nanotubes were immersed in an AgNO_3_ solution, enabling silver ions to be successfully loaded into the interior of the nanotubes, thereby enabling the preparation of a porous titanium material capable of releasing silver ions. The results showed that, in the early stages, this material demonstrated significant efficacy in inhibiting biofilm formation and reducing free bacteria. Ercan *et al*.’s [191] research showed that nanomodification of titanium surfaces has a significant effect on bacterial adhesion and growth. In particular, titanium nanotubes with a diameter of 80 nm exhibit stronger antibacterial properties. Furthermore, combining heat treatment and dual control of nanotube size can effectively reduce bacterial adhesion to titanium surfaces. It has been reported that the activity of inorganic metal ion antibacterial agents is dose dependent, but they are usually accompanied by dose-dependent cytotoxicity. To overcome this deficiency, Geng *et al*. [192] proposed adding Sr to silver-substituted HA coatings. Experiments revealed that the addition of strontium not only did not affect the antibacterial activity of silver but also significantly reduced its cytotoxicity. This improvement achieves a better balance between antimicrobial performance and biocompatibility, enhancing the potential for application as a bone implant. In addition, the addition of strontium helps control bone resorption and may have potential positive effects on the local treatment of osteoporosis (Figure 9A–D). However, the cell activity on the surface of the biocomposites is not as high as that on the HA surface. This issue can be addressed by balancing the doses of strontium and silver to achieve both excellent antibacterial performance and good biological activity. Additionally, Qiao *et al*. [193] deposited a hydroxyapatite (SSAgHA) coating containing Si, Sr and Ag on a TiO_2_ nanotube (TNT) array via an electrochemical deposition method. This codoped coating not only has superior biological properties compared with those of the HA and Ag-HA coatings but also has antimicrobial efficacy comparable to that of the Ag-HA coatings. These findings indicate that the doping of Sr and Si successfully counteracts the potential cytotoxicity of Ag ions. Importantly, it increases the expression levels of genes associated with osteogenesis, thereby addressing the drawback of poor cellular activity in Geng’s material.

Currently, the most widely used surface modification method for titanium-based implants is the layer-by-layer (LBL) method, which is based on the principle of supramolecular electrostatic assembly. This method involves the alternating adsorption of bioactive macromolecules with opposite charges on the substrate surface to form a self-assembled multilayer bioactive film [194, 195]. Yavari *et al*.'s [196] team successfully developed a multifunctional layered coating. First, they prepared porous commercial pure titanium (Cp-Ti) scaffolds via selective laser melting technology. Then, they immersed the scaffolds in gelatin/BMP-2 and chitosan/VAN solutions to achieve layered drug loading, resulting in porous titanium materials coated with multiple layers of BMP-2 and VAN. Experiments revealed that this novel biomaterial exhibits strong antibacterial properties and is capable of sustaining the release of VAN and BMP-2 for 2–3 weeks. The osteogenic differentiation capacity of BMSCs was enhanced, as manifested by a twofold increase in alkaline phosphatase activity and an up to fourfold increase in mineralization (Figure 9E–H). This study innovatively applied LBL technology to alternately coat 3D-printed porous titanium scaffolds with drugs and growth factors, thereby increasing the local drug concentration and sustaining the release of drugs. This has led to the design of a biomaterial with dual functions, promoting both antibacterial and osteogenic properties, which has great potential for application in the treatment of IBD.

Targeting the distinctive hyperglycemic and oxidative stress conditions present in diabetic patients, Xu *et al*. [197] proposed a composite functional coating for titanium-based implants, comprising MnO_2_ nanosheets embedded within a glucose/H_2_O_2_ dual-responsive hydrogel. Leveraging the cascade catalytic activity of MnO_2_ nanozymes, this system continuously consumes excess glucose and hydrogen peroxide in the local wound environment. This not only impairs bacterial energy metabolism and suppresses proliferation but also modulates the immunosuppressive milieu by promoting dendritic cell maturation and enhancing antigen presentation, thereby stimulating adaptive immune responses. Additionally, under pathological conditions, the hydrogel undergoes controlled degradation to release sonosensitizers that, upon ultrasound activation, generate ROS *in situ*, enabling precise and targeted antibacterial therapy. Manivasagam [198] and colleagues employed a combined thermochemical and silane modification approach to fabricate a microhierarchical/nanohierarchical superhydrophobic interface on titanium surfaces. This engineered interface displayed an exceptionally high-water contact angle, low surface energy and a negatively charged surface, effectively repelling similarly charged bacterial cells. As a result, bacterial adhesion and subsequent biofilm formation were significantly suppressed. Crucially, the superhydrophobic structure was integrally formed within the substrate rather than applied as an external coating, ensuring long-term interfacial stability and minimizing the risk of secondary contamination or immune responses associated with coating delamination. This design significantly enhanced the antibacterial properties, biocompatibility and overall safety of titanium implants in long-term clinical use. Fang *et al*.’s [199] team demonstrated the clinical advantages of 3D-printed, patient-specific titanium acetabular prostheses, which are custom-fabricated to precisely match individual bone defect morphologies. This structural congruence optimized intraoperative implant placement and reduced the need for repeated trimming, adjustments or temporary fixation—steps often necessary with conventional implants. As a result, surgical duration and anesthesia exposure were significantly reduced. The prostheses’ porous design and high conformity with host bone minimized micromotion and the formation of dead spaces, effectively lowering the risk of hematoma and fluid accumulation. Moreover, the high initial stability of the implants enabled early progressive weight-bearing, facilitating functional recovery and reducing postoperative immobilization time.

#### Magnesium-based biocomposites

Magnesium-based metals, with excellent biocompatibility and an ideal Young’s modulus close to that of natural cortical bone, are highly promising bone biomaterials. Unlike the aforementioned bioinert metals, magnesium-based metals have excellent bioactivity. They can promote osteogenesis, angiogenesis and neurogenesis while inhibiting osteoclast activity and inflammation, thereby accelerating the healing of bone defects [200–203]. Additionally, magnesium has a very low standard electrode potential, making it highly susceptible to corrosion in bodily fluids; as a result, it has high degradability and can eliminate the need for secondary surgery [204]. Mg^2+^ play crucial roles in human metabolism and are essential for bone synthesis. They can be excreted through the kidneys, implying that the metal ions released from implant degradation are not only harmless to the body but also contribute to bone regeneration [205]. However, magnesium metal has poor corrosion resistance and may degrade too quickly in complex physiological environments, resulting in mismatches with bone healing time and causing implant loosening. The gases produced and pH changes during the degradation process can cause local inflammation, greatly limiting the application of magnesium [206]. Currently, methods to improve the degradation rate of magnesium metal materials include alloying, surface modification and the addition of coatings [207].

**Figure 10:**
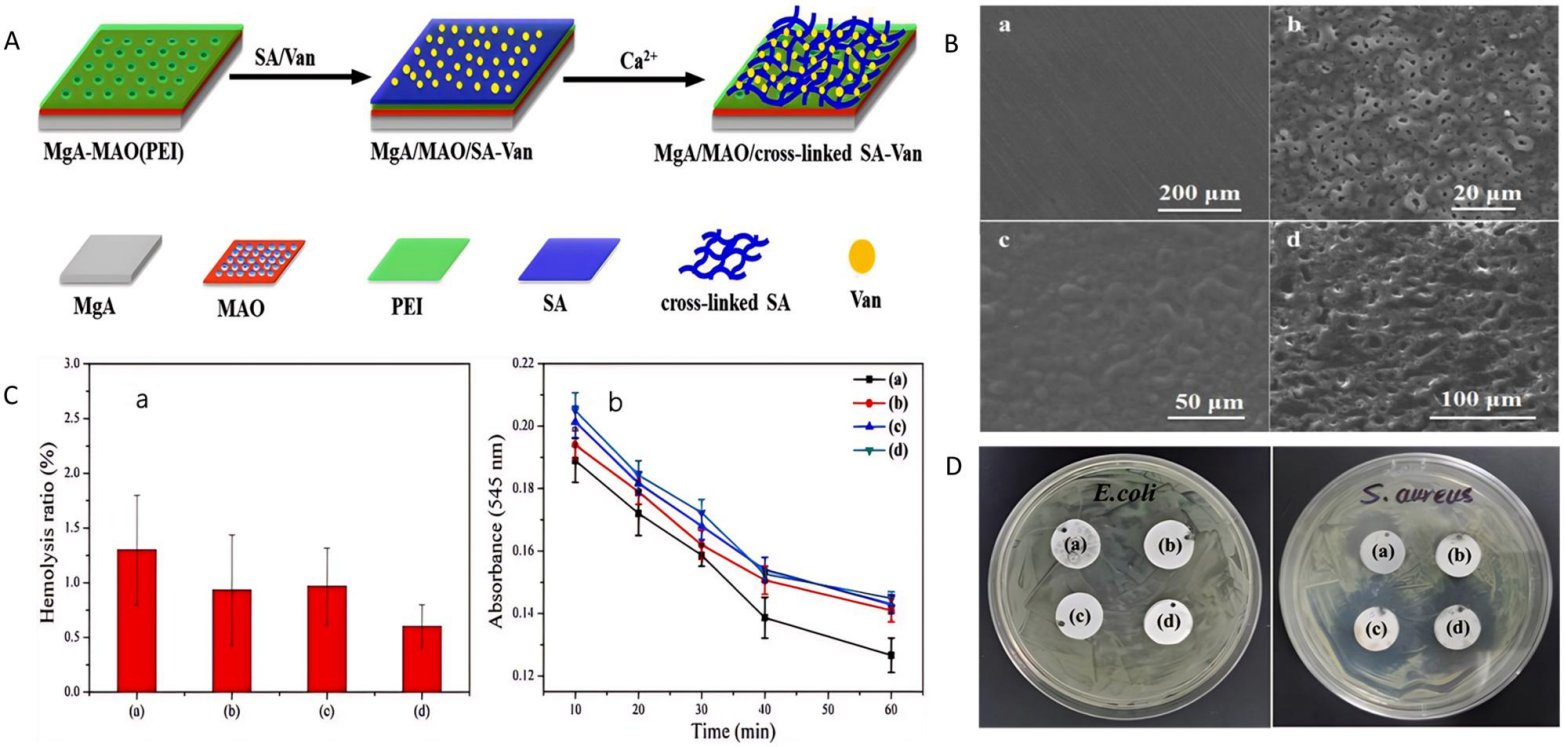
Synthesis of sodium alginate hydrogel composite coatings loaded with VAN on MAO-treated magnesium alloys. (**A**) Schematic diagram of the process of preparing a sodium alginate hydrogel coating loaded with vancomycin on the surface of magnesium alloy treated with micro-arc oxidation. (**B**) Surface SEM images of MgA (a), MgA/MAO (b), MgA/MAO/SA-Van (c) and MgA/MAO/crosslinked SA-Van (d). (**C**) Haemolysis rate (left) and dynamic clotting time (right) of different coated samples. (**D**) Antibacterial effects of different coated samples on *E. coli* and *S. aureus*. Reproduced with permission from Ref. [210], Copyright 2020, Elsevier.

Among these methods, chemical surface modification is considered more reliable, as it prevents the coating from peeling or wearing off during the injection process [208]. Microarc oxidation (MAO) is a promising chemical modification technology that results in excellent adhesion to metal substrates. The formation of a highly adhesive porous structure on the substrate surface effectively enhances the corrosion resistance of magnesium-based metals [209]. Du *et al*. [210] prepared sodium alginate hydrogel composite coatings loaded with VAN on magnesium alloys treated with MAO. The results showed that not only was the corrosion resistance of the magnesium alloys improved, but also that they exhibited excellent antibacterial properties and good blood compatibility (Figure 10).

**Figure 11:**
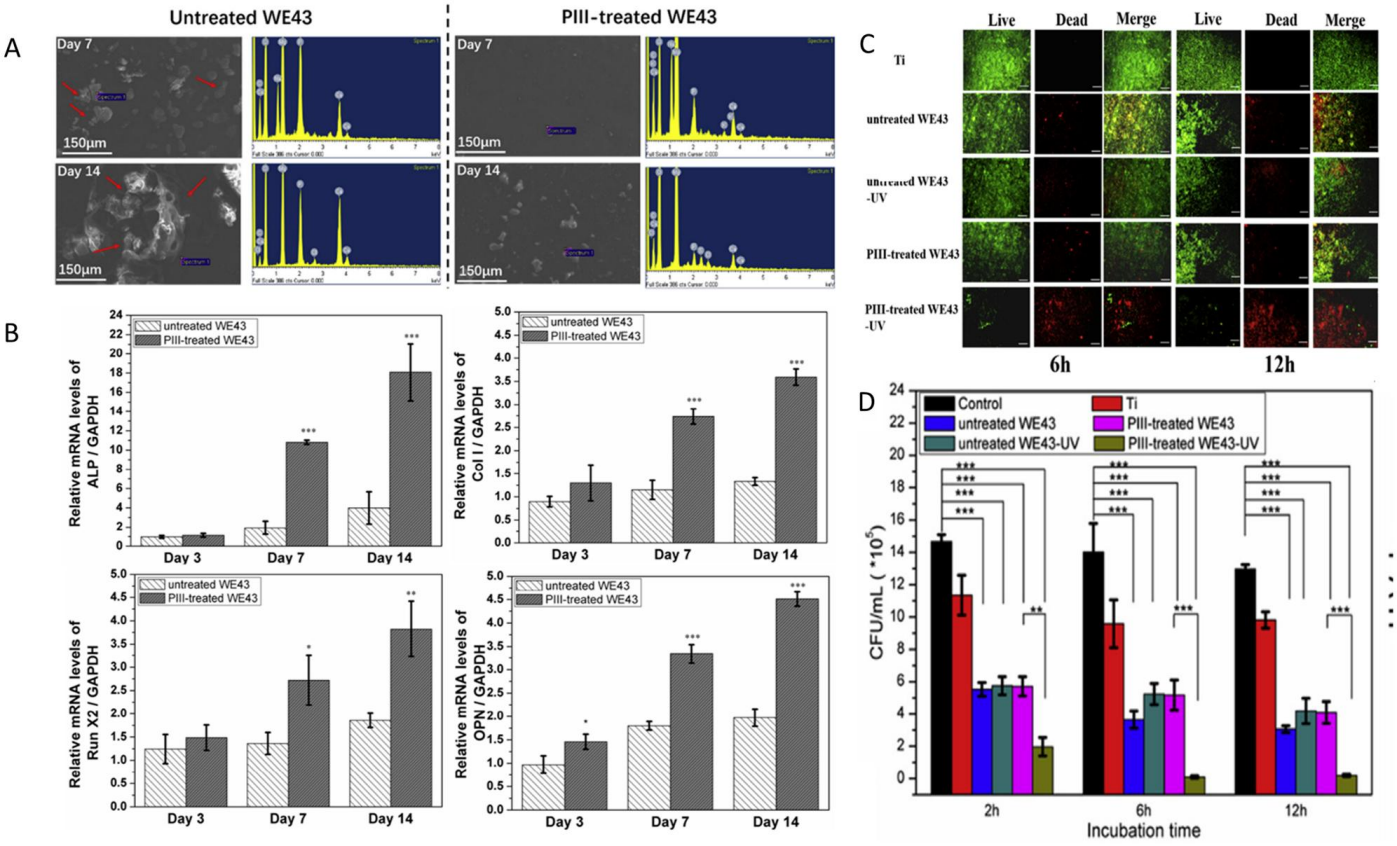
Synthesis of functionalized TiO_2_/Mg_2_TiO_4_ nanolayers on WE43 magnesium implants. (**A**) Surface morphology and EDS spectra of untreated and PIII-treated WE43 samples after immersion in SBF solution at 37°C for 7 and 14 days. (**B**) Osteogenic gene expression in the unprocessed and PIII-treated WE43 groups. (**C**) Live/Dead fluorescence images of *Staphylococcus aureus* suspension on the surface of Ti, untreated WE43, untreated WE43-UV, PIII-treated WE43 and PIII-treated WE43-UV samples. (**D**) Adherent bacterial counts for each group determined by the plate count method after incubation at 37°C for 2, 6 and 12 h. Reproduced with permission from Ref. [211], Copyright 2019, Elsevier.

In recent years, researchers have reported that the corrosion of magnesium metal in an *in vitro* environment leads to an increase in the pH value of the culture medium and a corresponding increase in the Mg^2+^ concentration, which in turn inhibits bacterial growth. Lin *et al*.’s [211] team used plasma immersion ion implantation (PIII) technology to prepare functionalized TiO_2_/Mg_2_TiO_4_ nanolayers on WE43 magnesium implants. Research indicates that TiO_2_/Mg_2_TiO_4_ nanolayers significantly enhance the corrosion resistance and osteoblast differentiation ability of magnesium alloy implants, while also exhibiting antibacterial properties under ultraviolet light. This demonstrates their potential for clinical application in the treatment of bone defects (Figure 11). However, unmodified pure Mg and its alloys do not have sufficient mechanical strength to provide early support for high-load-bearing bone defects. Extensive research has shown that adding alloying elements, such as zinc, neodymium, calcium and germanium, to magnesium can effectively improve its mechanical properties, biocompatibility and corrosion resistance, while also reducing hydrogen gas production and thereby minimizing inflammatory responses [212]. Recent research has focused on Mg-5%Zn-based alloys, particularly those with excellent mechanical properties and corrosion resistance imparted by zinc inclusions. To enhance the mechanical and electrochemical properties of this alloy, scholars have introduced and studied several alloying elements, including rare earth elements, in detail. Elkaiam *et al*. [213] obtained a new type of alloy by adding different concentrations of neodymium to a Mg-5%Zn-0.13%Y-0.35%Zr alloy. Experiments revealed that when the Nd content was 2%, the alloy exhibited optimal mechanical properties, including high strength and good ductility. This was mainly due to the addition of Nd, which altered the microstructure of the alloy and promoted the formation of specific secondary phases.

To mitigate the heightened risk of postoperative infection in diabetic patients, Lian *et al*. [214] developed a sprayable hydrogel dressing incorporating magnesium–gallic acid metal–organic frameworks (Mg-GA MOFs). The sustained release of gallic acid effectively scavenges ROS, reduces oxidative stress and promotes the polarization of macrophages from the pro-inflammatory M1 phenotype to the reparative M2 phenotype, thereby facilitating the transition from the inflammatory to the proliferative phase of wound healing. Concurrently, the gradual release of Mg^2+^ ions enhance endothelial cell migration and regeneration, contributing to the reconstruction of the neurovascular network. The integration of HACC further imparts antibacterial properties to the hydrogel, providing robust protection against infection. The sprayable nature of the formulation allows for rapid film formation across the wound surface, offering convenient clinical application and sustained microenvironmental regulation for multifunctional wound repair in diabetic settings. Zhang *et al*. [215] designed an iron-based composite coating with switchable wettability for magnesium alloy implants. Thermal treatment induced partial disruption of Fe–OH bonds, introducing surface defects that transitioned the coating from a hydrophilic to a superhydrophobic state. This transformation significantly enhanced the coating’s self-cleaning and antifouling capabilities, effectively minimizing bacterial adhesion and reducing the risk of biofilm development.

#### Tantalum-based biocomposites

Compared with first-generation and second-generation metallic bone repair materials, tantalum (Ta) has excellent biocompatibility, chemical stability, favorable osteogenic and angiogenic properties and superior mechanical performance. Ta has a high affinity for oxygen, forming a passivated oxide layer (Ta_2_O_5_) on its surface, which facilitates the formation of bone-like HA coatings and studies have indicated that it also exhibits antibacterial properties [216, 217]. Therefore, tantalum has very low solubility at all pH values and potentials, reducing the occurrence of local inflammation caused by corrosion products [218]. Although tantalum has several excellent properties, the elastic modulus of solid tantalum is still significantly greater than that of natural cortical bone and cancellous bone in the human body. The difference in elastic modulus may cause a stress shielding effect, which may ultimately lead to loosening and detachment of the implant [219]. To address the performance issues of tantalum materials, researchers have made improvements by adding other materials or optimizing the preparation process. For illustration, Huang *et al*. [220] used SLM technology to prepare titanium-tantalum alloys. The experimental results revealed that the alloy containing 30% tantalum had the lowest elastic modulus and the highest Young’s modulus, and its ultimate tensile strength was comparable to that of the other alloys, significantly improving the mechanical properties of the biocomposites.

With advancements in manufacturing technology, the development of porous tantalum structures has addressed the issue of excessive weight in dense tantalum, while reducing its elastic modulus and thereby mitigating the stress shielding effect. For instance, Zhao *et al*. [221] used tantalum oxide powder to prepare porous tantalum scaffolds with mechanical properties similar to those of human cancellous bone. The porous structure of tantalum creates a favorable environment for oxygen and nutrient transport, promoting the adhesion and proliferation of cells [222]. However, the high melting point and affinity for oxygen make the preparation of porous tantalum difficult. Moreover, porous tantalum scaffolds prepared via traditional methods are not only expensive but also have irregular pore structures and cannot be customized. The development of 3D printing technology offers greater flexibility in designing porous tantalum scaffolds, allowing for more effective replication of the microstructure of bone tissue, which benefits the proliferation of osteoblasts and enhances the integration of surrounding bone tissue. Personalized porous tantalum scaffolds can be functionalized through surface modification and incorporation of drugs to impart osteogenic and antibacterial properties, meeting specific clinical needs. For example, Ma *et al*. [223] utilized the strong adhesive properties of a polydopamine (PDA) coating to uniformly load Mg^2+^ onto the surface of a 3D-printed porous tantalum scaffold (Ta-PDA-Mg). The experimental results revealed that the Mg-doped scaffold promoted better cell adhesion, osteogenic differentiation and angiogenesis.

Research has shown that tantalum can induce high expression of osteogenic genes and certain cytokines, while reducing the expression of osteoclast-related genes, thereby promoting the proliferation, differentiation and mineralization of osteoblasts [224]. Although the oxide layer on the surface of tantalum has certain antibacterial properties, this passive antibacterial effect is insufficient in cases of severe infection or in environments with drug-resistant bacteria. The use of tantalum alone is insufficient to effectively inhibit the proliferation of pathogens in infected areas. Therefore, to meet the needs of clinical applications for treating IBD, physical and chemical methods can be used to modify the properties of tantalum, enabling it to exhibit both powerful antibacterial and osteogenic functions.

**Figure 12:**
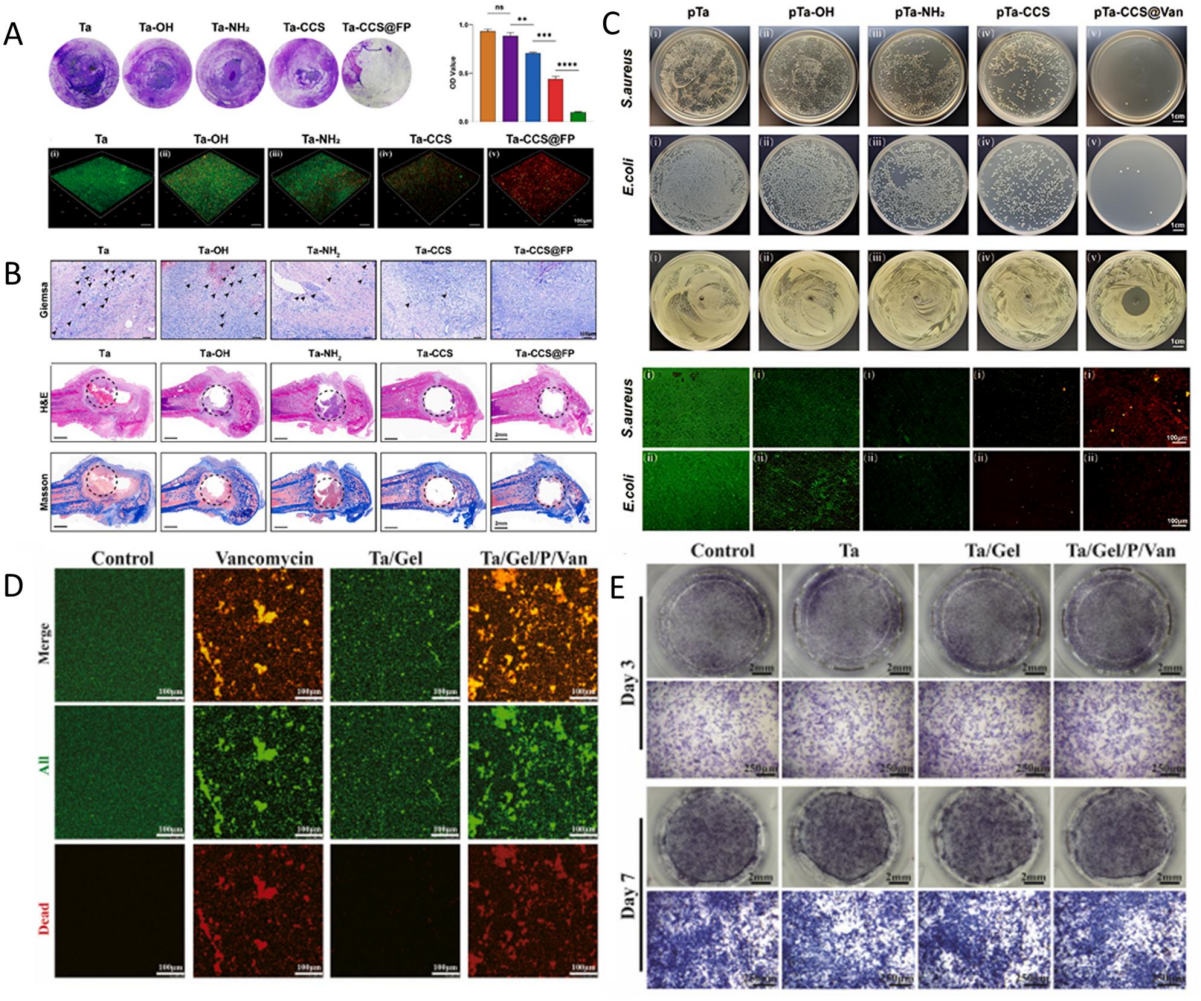
Analysis of multifunctional tantalum-based biocomposites. (**A**) The effect of Ta-CCS @FP scaffolds on biofilm formation and its mechanism of action. (**B**) Giemsa staining, H&E staining and Masson staining results of the bone tissue surrounding different scaffold materials. Reproduced with permission from Ref. [225], Copyright 2025, Elsevier. (**C**) Antibacterial performance of Ta scaffolds with different surface modifications in antibacterial experiments. Reproduced with permission from Ref. [226], Copyright 2022, American Chemical Society. (**D**) Live/dead bacterial staining images of Ta/Gel/PLGA/Van composite scaffold. (**E**) ALP staining of Ta/Gel/PLGA/Van composite scaffold. Reproduced with permission from Ref. [227], Copyright 2023, Elsevier.

For example, Liu *et al*. [225] designed a fusion peptide-grafted chitosan coating (Ta-CCS@FP) on the surface of a porous tantalum scaffold, which has dual anti-infection and bone-promoting functions. These results indicate that the Ta-CCS@FP coating effectively enhances antibacterial properties through the synergistic action of fusion peptides (HHC36 and the QK peptide), significantly reducing the adhesion and growth of *S. aureus* and *E. coli* and inhibiting biofilm formation. Moreover, the coating promoted osteogenesis and angiogenesis in BMSCs and HUVECs, thereby enhancing the bonding between the implant and bone tissue through the coupling of these two processes (Figure 12A and B).

Studies on the use of porous AM-Ta to load antibiotics are limited, and achieving prolonged, continuous release of antimicrobial drugs without the toxicity caused by burst release remains a challenge. Liu *et al*. [226] proposed a 3D printing method for surface modification of porous tantalum by adding VAN and carboxymethyl chitosan composite coatings to improve the antibacterial and osteogenic properties of the tantalum material. They reported that the pTa scaffold with the loaded coating effectively killed attached bacteria in the early stage and inhibited biofilm formation, demonstrating significant antimicrobial properties. The coating was also biocompatible and did not alter the pore structure or specific surface area of the tantalum material, supporting the adhesion and proliferation of BMSCs and promoting the expression of osteogenesis-related genes (Figure 12C). Qian *et al*. [227] used AM technology to fabricate pTa scaffolds, loaded VAN in PLGA microspheres and combined them with GelMA hydrogels to form a system that can continuously release antibiotics. This method facilitates sustained-drug release and avoids the toxicity and short-lived effects caused by rapid drug release, effectively improving antibacterial efficacy and reducing systemic toxicity. It also created a favorable microenvironment for the proliferation and osteogenic differentiation of BMSCs, further enhancing the osteogenic ability of the composite scaffold (Figure 12D and E).

To further reduce the incidence of implant-associated infections (IAI) in clinical settings, Bandyopadhyay [228] fabricated Ti–Ta–Cu alloys using laser powder bed fusion technology. The incorporation of Cu^2+^ ions imparted intrinsic antibacterial activity via Cu^2+^–surface interactions, significantly inhibiting bacterial adhesion and biofilm formation. The alloy exhibited enhanced mechanical performance, including improved shear strength and fatigue resistance, thereby ensuring long-term implant stability and reducing intraoperative handling time. Additionally, Ti_3_Al_2_V–Cu alloys demonstrated superior early-stage osseointegration, contributing to accelerated postoperative recovery and a reduced risk of complications.

### Organic-based biocomposites

The use of adequate amounts of antibiotics for the full course of treatment, which is a lengthy and time-consuming process, is necessary for treating IBD. Traditional drug delivery methods have many drawbacks, such as unnecessary toxic side effects caused by long-term repeated use of antibiotics on other parts of the body and a lack of drug targeting, which can lead to reduced concentrations before the antibiotics reach the site of the disease, and thus, cause drug resistance and an uncontrollable release rate. Therefore, an increasing number of scholars are committed to developing more effective drug delivery systems that incorporate antimicrobial agents into implant materials as an *in situ* treatment method to address these limitations [229]. Local drug delivery systems are generally constructed using biodegradable materials, for example, polymers, lipid materials and nanomaterials. These carriers provide stable drug encapsulation and sustained release, making drug treatment more efficient and precise while reducing systemic side effects [230, 231]. Although these materials can preliminarily meet the treatment needs of IBD through their own physical and chemical properties, in actual applications, their main function is often not as a scaffold substrate but rather as a drug delivery system to address complex pathological microenvironments. Therefore, this section focuses on multifunctional carrier materials for drug delivery systems that precisely deliver active molecules, for example, antibiotics and growth factors, breaking through the performance bottlenecks of single materials to achieve synergistic regulation of infection prevention, osteogenesis and biodegradability.

#### Hydrogel

Hydrogels are highly hydrated three-dimensional network polymers that can be molded into different sizes and shapes as needed [232, 233]. Similar to the extracellular matrix, they can retain large amounts of water through absorption and swelling. Owing to its unique properties, which are highly similar to those of human tissue, and its excellent biocompatibility and degradability, it has been widely researched and applied in the field of biomedicine [234]. Hydrogels can be classified into natural hydrogels, synthetic hydrogels and composite hydrogels on the basis of their source. Natural hydrogels are generally considered to have better biocompatibility and bioactivity, whereas synthetic hydrogels offer more tunable mechanical and degradation properties [235]. In recent years, a wide variety of hydrogels made from different polymers, including chitosan, gelatin, alginate, hyaluronic acid and polyethylene glycol, have been developed. Each type of hydrogel has its own unique advantages and limitations [236]. Hydrogels also exhibit high tunability, allowing researchers to precisely control the release rate of molecules by adjusting the network size or degradation rate of the hydrogel. This property makes hydrogels highly promising for use in drug delivery applications.

Unlike other carriers, the use of hydrogels as drug carriers significantly expands the range of antibiotics that can be selected, allowing multiple antibiotics to be loaded into a single hydrogel. Numerous experiments have demonstrated that the sustained-release time of antibiotics *in vitro* extends from less than 96 h to more than 6 weeks. In contrast, there are relatively few reports on the release time *in vivo*; however, based on existing research data, this time range also appears to be between 72 h and more than 6 weeks [237, 238]. They also compared the efficacy of antibiotic-loaded PMMA bone cement (ACR) and an antibiotic-loaded hydrogel (HA-pNipam) in treating chronic MRSA orthopedic device-related infections (ODRIs) in sheep. The experimental results revealed that the two treatments were equally effective, and the data indicated that antibiotics could be continuously released from the HA-pNipam hydrogel for more than 10 days, with local concentrations higher than the MIC.

Antimicrobial peptides (AMPs) are active peptides that are widely found in nature and form part of the body’s natural immune defense system. Extensive research has demonstrated that AMPs exhibit powerful antimicrobial activity against a variety of microorganisms, including biofilms, while preventing the development of bacterial resistance and exhibiting no cytotoxicity [239]. When administered systemically, AMPs typically exhibit poor stability in the gastrointestinal tract and other body fluids, resulting in poor absorption and distribution efficiency, which reduces their bioavailability [239]. Therefore, direct delivery of AMPs to bone infection sites via hydrogels is an effective clinical application strategy. Yang *et al*. [240] designed a RADA16 self-assembling hydrogel to deliver AMPs. This study revealed that this hydrogel could stably and continuously release AMPs for up to 28 days, effectively inhibiting the growth of *S. aureus* while promoting the proliferation of BMSCs. The RADA16-AMP hydrogel has the potential to serve as a dual-function material with antibacterial and bone regeneration properties for the treatment of IBD.

**Figure 13:**
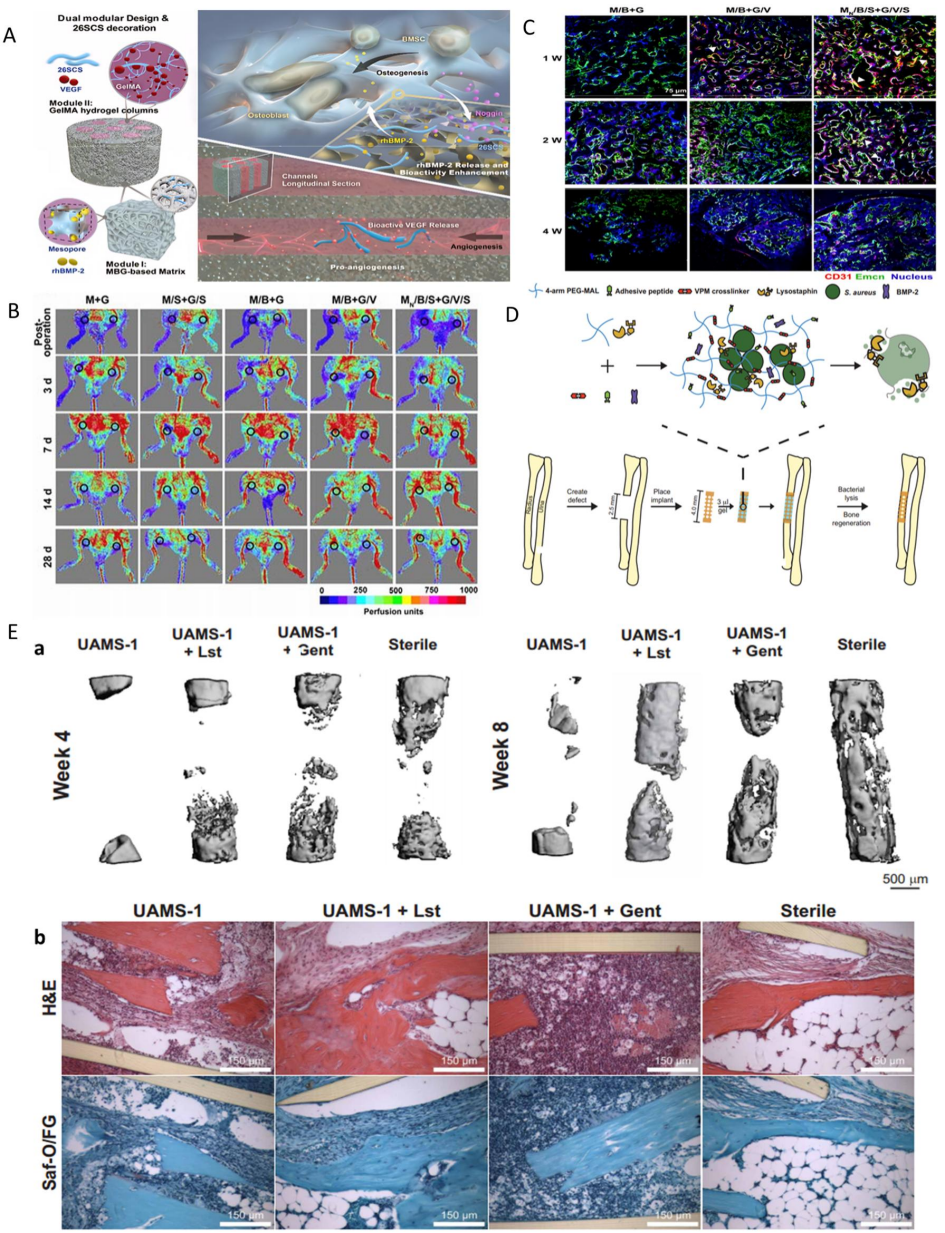
Analysis of multifunctional hydrogel delivery systems. (**A**) Design of the 26SCS functionalized dual-modular scaffold for GFs delivery to enhance bone regeneration. (**B**) Representative laser doppler scans at Day 3, 7, 14 and 28 after ischemia induction and implantatio. (**C**) Images of bone vessels stained for CD31 and Endomucin. Reproduced with permission from Ref. [241], Copyright 2020, Elsevier. (**D**) Lysostaphin and BMP-2 co-delivery to a critical-sized segmental bone defect. (**E**) UAMS-1 infection defects treated with BMP-2–loaded lysostaphin-delivering hydrogels significantly improve bone repair. (a) Quantification of bone volume from CT imaging at 4 and 8 weeks after implantation. (b) Mouse radii were sectioned and stained with H&E and safranin-O/fast green (Saf-O/FG). Reproduced with permission from Ref. [27], Copyright 2019, American Association for the Advancement of Science.

Bioactive factors, such as cytokines and growth factors, play crucial roles in new bone formation by regulating cell proliferation, migration, differentiation and extracellular matrix synthesis through precise molecular mechanisms. Tang *et al*. [241] developed a dual-module scaffold composed of mesoporous BG (MBG, module I) and GelMA hydrogel columns (module II), which was functionalized with 2-N, 6-O-sulphated chitosan (26SCS) to achieve the differentiated release of BMP-2 and VEGF. *In vitro* and *in vivo* experiments confirmed that this specific GF delivery method and 26SCS decoration can effectively coordinate the two interrelated processes of osteogenesis and angiogenesis and enhance both processes (Figure 13A–C). The simultaneous incorporation of BMP and antimicrobial agents can promote bone growth during the treatment of bone infections, which has important clinical value in the treatment of IBD. Previous studies have shown that antimicrobial agents added to hydrogels do not significantly interfere with the release of BMP-2, and antibiotics loaded in this manner do not inhibit the role of BMP-2 in repairing bone defects [242].

Staphylolytic enzymes are endopeptidases produced by certain species of staphylococci that have the ability to specifically break down the cell walls of *S. aureus* and other staphylococci. Although staphylococcal enzymes have shown great potential in laboratory studies and animal models, their use in clinical applications still faces some challenges, for example, enzyme stability, safety in humans and efficacy. To overcome these challenges, researchers are developing improved versions of staphylococcal enzymes and more effective delivery systems to increase their potential for use in antimicrobial treatments. The Johnson *et al*. [27] team designed a polyethylene glycol 4MAL hydrogel as a delivery platform for BMP-2 and *S. aureus*. In a mouse model, researchers created a 2.5-millimeter nonhealing bone defect. The experimental results showed that this lysozyme-delivery hydrogel could completely eradicate *S. aureus* infection within 7 days and promote the regeneration of segmental bone defects, providing an effective strategy for treating complex IBD (Figure 13D and E).

#### Microsphere

Microspheres are tiny spherical particles, typically ranging in diameter from a few micrometers to several hundred micrometers. Compared with other materials, they are widely used in biomedicine for drug delivery because of their specific targeting properties and ability to achieve slow drug release [243]. In terms of drug delivery, the inherent spherical structure of microspheres results in a larger surface area than 3D-printed scaffolds or electrospun fibers do, thereby improving drug loading efficiency and increasing the area of drug contact. Compared with microcapsule-type drug sustained-release systems, the mechanism of drug release in microsphere systems relies primarily on the degradation process of the carrier material. This difference prevents the excessive release of drugs in the early stages due to the rupture or dissolution of the outer capsule. Additionally, in the later stages of drug release, the microsphere-based sustained-release drug delivery system can ensure that drugs are released at a sustained and constant rate through the degradation of the carrier material, effectively preventing the issue of slowed drug diffusion rates caused by a decrease in the drug content within the microspheres [244]. They can be made from a variety of materials, including natural polymers, synthetic polymers and other biocompatible materials. The choice of material depends on the intended use of the microspheres, including their biocompatibility, biodegradability, mechanical properties and chemical stability in specific applications [245]. Microspheres can be divided into two main categories: microcapsules and micromatrices. Microcapsules enclose substances within independent spaces via wall materials, making them suitable for protecting and controlling the release of their contents. In contrast, substances in micromatrices are dispersed throughout the matrix, making them suitable for the gradual release of active ingredients. These two structures determine their different applications and release mechanisms [246].

**Figure 14:**
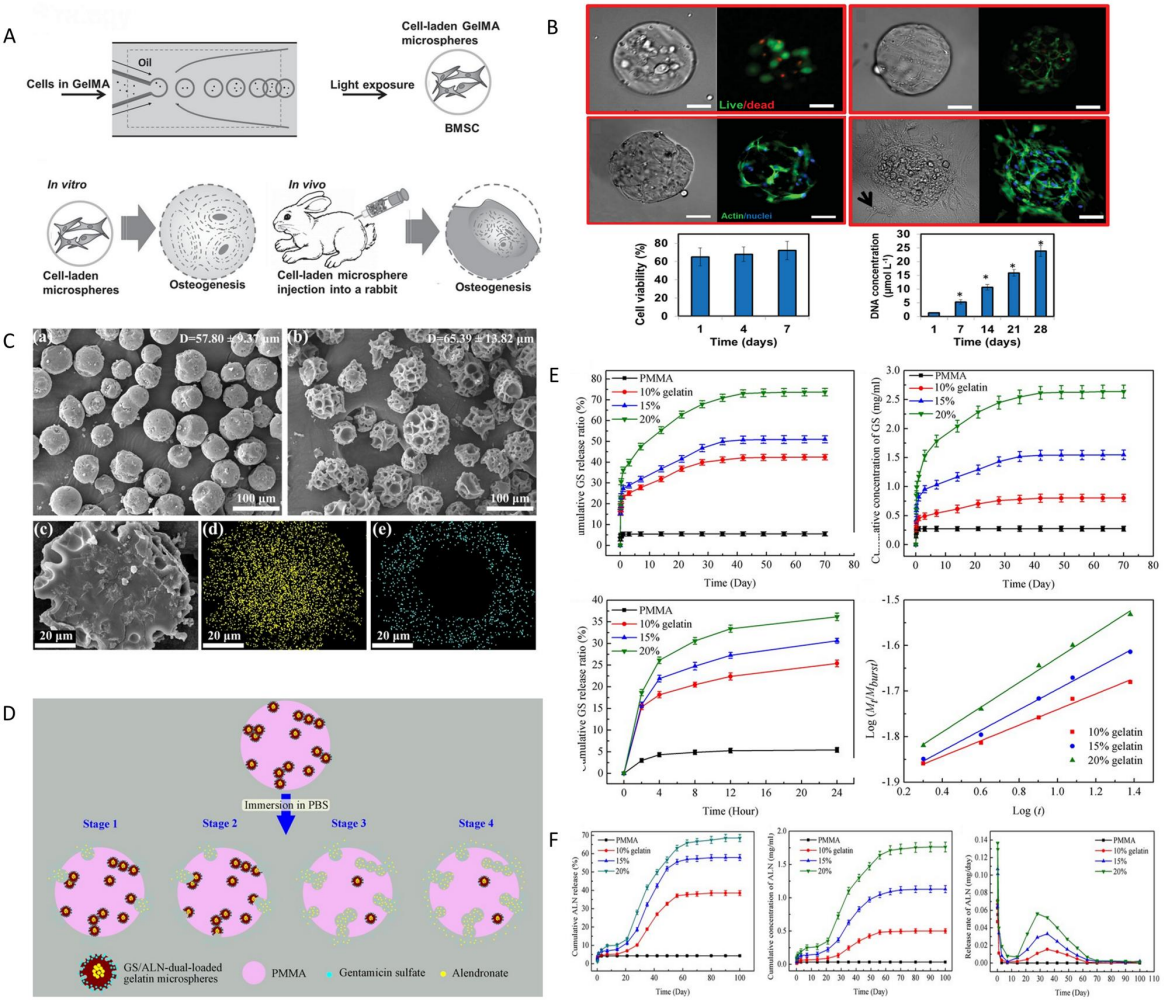
Analysis of multifunctional microsphere delivery systems. (**A**) Schematic diagram of fabrication of BMSC-laden GelMA microspheres and its application for osteogenesis and regeneration of injured bones. (**B**) Viability, spreading and proliferation of BMSCS encapsulated in GelMA microspheres. Reproduced with permission from Ref. [248], Copyright 2016, Wiley. (**C**) SEM images of (a) ALN-loaded gelatin microspheres; (b) GS/ALN-dual-loaded gelatin microspheres; (c) SEM image of a microsphere cross-section; and EDS mapping of (d) elemental phosphorus and (e) sulfur (blue dots). (**D**) Schematic diagram of ALN release during the different stages. (**E**) GS release profile. (**F**) ALN release profile. Reproduced with permission from Ref. [250], Copyright 2021, Royal Society of Chemistry.

In recent years, MSCs have been widely studied as seed cells in bone tissue engineering. These cells can grow and differentiate into osteoblasts to promote the formation of new bone tissue. Numerous studies have shown that MSCs can also promote vascular regeneration, reduce cell apoptosis and inhibit the occurrence of inflammatory responses [247]. Zhao *et al*. [248] used microfluidic technology to encapsulate BMSCs and BMP-2 in GelMA microspheres, ultimately preparing injectable osteogenic tissue structures. The results showed that the microspheres could support cell survival and migration from the interior to the surface (Figure 14A and B).

In bone tissue engineering, microspheres can also serve as carriers for drugs and active factors, thereby effectively treating IBD. The injectability of microspheres enables them to fill bone defects of various sizes, reducing surgical invasiveness and the risk of infection. The micron-sized gaps formed between the microspheres promote cell penetration and nutrient metabolism. Compared with traditional scaffolds, this approach enhances cell permeability and nutrient exchange, creating favorable conditions for angiogenesis and thereby optimizing the bone regeneration process [249]. The design and synthesis of microspheres are a highly customized process that needs to be optimized according to specific clinical needs. Chen *et al*. [250] designed a GS/ALN-gelatin dual-loaded modified PMMA bone cement (GAPBC) by encapsulating gentamicin sulfate and alendronate phosphate into gelatin microspheres, and then, dispersing these microspheres in a PMMA bone cement matrix. The experimental results showed that GAPBC achieved the orderly release of GS and ALN, with GS playing a major role in the first 24 h and ALN release dominating after 3 weeks of PBS immersion. By adjusting the microsphere content, the release rates of GS and ALN were controllable. *In vitro* experimental results revealed that GAPBC is significantly superior to traditional PMMA bone cement in promoting cell proliferation and adhesion (Figure 14C–F). Guo *et al*. [251] prepared a novel drug delivery system. This drug carrier has a drug loading efficiency of up to 70–75%, which is significantly greater than the drug loading efficiency of traditional hydroxyapatite particles. Research has shown that it can significantly reduce bacterial adhesion and prevent biofilm formation, especially against *S. aureus*. Biocompatibility testing using hBMSCs as a cell model demonstrated that gentamicin-loaded MCHMs exhibit the same excellent biocompatibility as HAPs. Furthermore, the gentamicin released from MCHMs had no toxic effects on hBMSCs. Therefore, gentamicin-loaded MCHMs can serve as an effective local drug delivery system for the treatment of IBD.

#### Polymethyl methacrylate

Polymethyl methacrylate (PMMA) is a linear, transparent thermoplastic polymer synthesized through free-radical polymerization of methyl methacrylate monomers. Due to its superior optical characteristics, it is commonly referred to as ‘organic glass’ [252]. The presence of polar ester groups within its molecular structure imparts a combination of high transparency, elevated refractive index, mechanical robustness and resistance to weathering, ensuring long-term stability under UV radiation and in complex environmental conditions. Compared to inorganic materials, PMMA possesses notable advantages such as low density, high plasticity and excellent processability. These properties enable its fabrication into intricate geometries through techniques such as injection molding, extrusion and 3D printing, thereby broadening its applicability in biomedical fields. In tissue engineering and regenerative medicine, PMMA is widely utilized in bone cements, dental prostheses and drug delivery systems due to its biocompatibility and chemical inertness. Nonetheless, its poor biodegradability and mechanical mismatch with native bone tissue continue to limit its suitability for long-term implantation and use in dynamic load-bearing environments [89].

Furthermore, PMMA is bioinert and lacks the capacity to form chemical bonds with surrounding bone tissue, resulting in weak interfacial integration. This insufficient bonding often leads to micromotion during daily activities, which can generate particulate wear debris that induces osteolysis, potentially culminating in aseptic loosening or displacement of cemented implant [253]. Therefore, recent studies have focused on enhancing PMMA’s mechanical performance, interfacial compatibility and bioactivity by incorporating inorganic fillers or biodegradable polymers such as hydroxyapatite (HA), carbon nanotubes and natural polymers. These modifications aim to extend PMMA’s clinical utility, particularly in the treatment of infectious bone defects [254]. Wang *et al*. [255] incorporated MgAl layered double hydroxide (LDH) nanosheets into PMMA bone cement to produce a PMMA & LDH composite with improved osteointegration. Experimental results revealed that the incorporation of LDH significantly reduced the peak polymerization temperature, thereby minimizing the risk of thermal injury during surgery. Additionally, the composite exhibited moderated mechanical properties that helped alleviate stress shielding, a common cause of osteolysis. Mg^2+^ released from the LDH phase further promoted osteogenesis, as evidenced by both *in vitro* and *in vivo* models. The composite also demonstrated rapid polymerization and immediate setting characteristics, enabling timely fixation of prosthetic components and facilitating faster postoperative recovery and earlier return to normal function.

For over four decades, PMMA has been widely regarded as the gold standard biomaterial for local antibiotic delivery in orthopedic applications. It has been extensively employed to deliver high concentrations of antibiotics directly to infection sites and to occupy dead spaces resulting from debridement procedures, thereby aiding in the prevention and treatment of bone infections. Antibiotic-loaded PMMA bone cement has found clinical utility across various scenarios, including total knee and hip arthroplasty, nonweight-bearing orthopedic revision surgeries, temporary antibiotic-loaded spacers and antibiotic beads for infection management. Despite its widespread use, PMMA’s polymerization is highly exothermic, and the elevated temperatures generated during curing are associated with several adverse effects, including thermal necrosis of adjacent tissues, compromised local blood circulation and the potential formation of fibrous membranes at the bone–cement interface. Multiple strategies have been proposed to reduce PMMA’s peak exothermic temperature, such as decreasing ambient room temperature, pre-cooling cement components before mixing and using lower molecular weight PMMA powders [256]. However, these methods have shown only limited efficacy. To address this limitation, Yang *et al*. developed a PMMA composite material for vertebroplasty (VP) and balloon kyphoplasty (BKP) by incorporating phase-change material microcapsules (PCMc). These microcapsules effectively absorbed excess heat during polymerization, thereby reducing thermal injury. The inclusion of 20% PCMc significantly decreased the peak exothermic temperature, extended the setting time and slightly lowered the compressive strength and elastic modulus of the cement. *In vitro* studies confirmed the composite’s biocompatibility with L929 fibroblasts [257].

**Figure 15:**
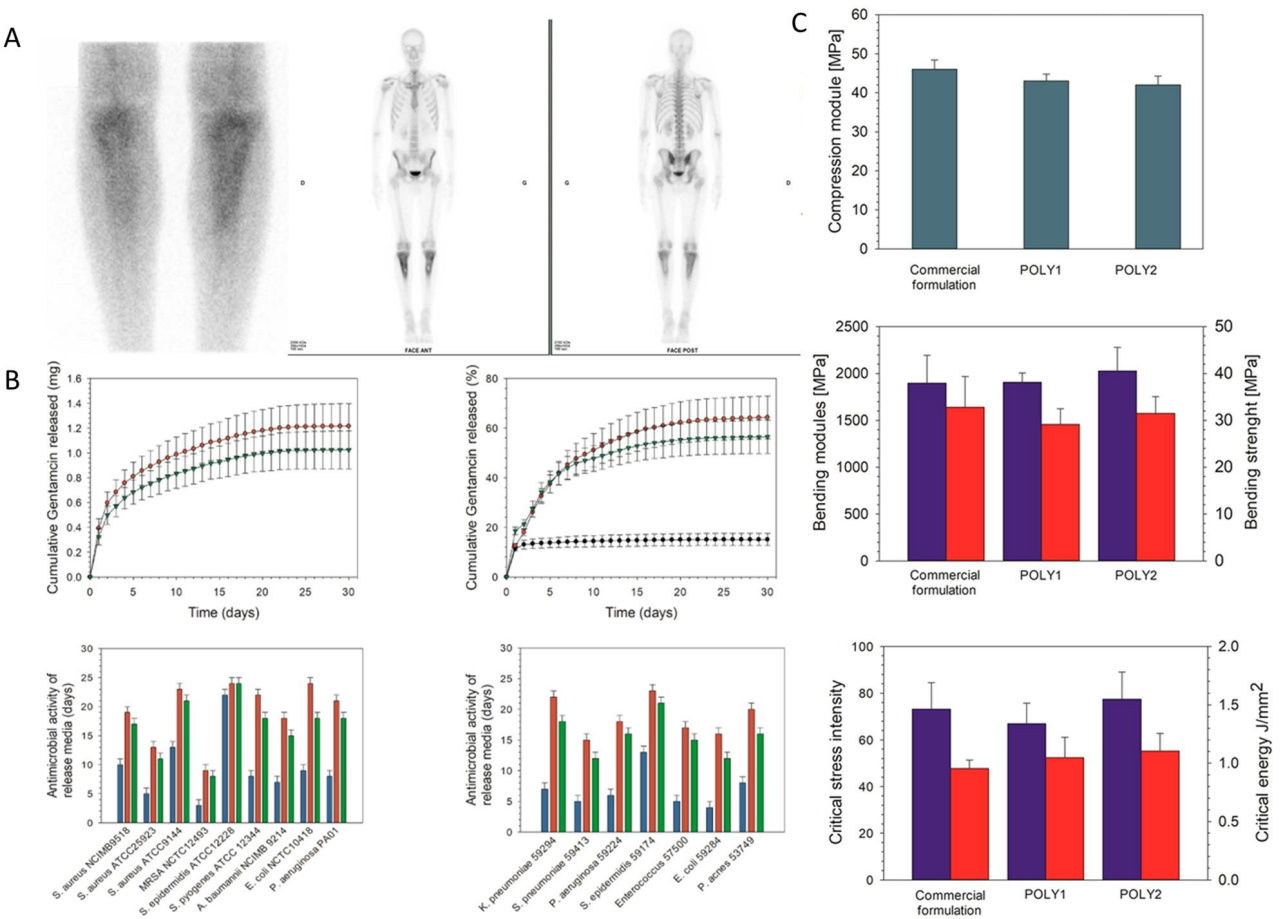
Analysis of antibiotic-loaded PMMA treatment for IBD. (**A**) Planar images of 99mTc bone scan of a 21-year-old man who underwent surgery for bilateral osteomyelitis of the tibias in Russia 9 months ago. Reproduced with permission from Ref. [259], Copyright 2022, Wolters Kluwer. (**B**) Incorporating silica nanocarriers into PMMA bone cement significantly extended gentamicin release. (**C**) The introduction of nanocarriers did not adversely affect the key properties of the bone cement. Reproduced with permission from Ref. [261], Copyright 2019, American Chemical Society.

Because PMMA polymerizes at high temperatures, only thermally stable antibiotics are suitable for incorporation. Preferred agents include aminoglycosides—such as streptomycin, gentamicin, amikacin and tobramycin—and cephalosporins [258]. Février *et al*. [259] reported a clinical case in which gentamicin-loaded PMMA beads were successfully used to manage bone defects caused by chronic osteomyelitis. This local delivery system achieved antibiotic concentrations at the infection site significantly higher than those attainable through systemic administration, thereby ensuring improved therapeutic outcomes (Figure 15A).

However, the addition of antibiotics to PMMA can compromise its mechanical integrity. Antibiotic molecules may interfere with the polymerization process, particularly liquid formulations, which can severely disrupt curing. For example, Hsieh *et al*. [260] observed a 37% reduction in compressive strength when liquid gentamicin was added to Simplex bone cement. Consequently, only crystalline antibiotics are generally recommended and should be incorporated into the powder component prior to mixing. In this context, Perni *et al*. [261] reported a novel drug delivery approach by loading gentamicin onto silica nanocarriers and encapsulating them via LBL self-assembly using poly(β-amino ester) (PBAE) and alginate. These nanocarriers were embedded within PMMA bone cement to extend antibiotic release and improve long-term management of infectious bone defects. This system prolonged gentamicin release to approximately 30 days—substantially longer than the ∼6 days typically observed with conventional antibiotic powders. It also exhibited broad-spectrum antibacterial activity against pathogens commonly associated with prosthetic joint infections. Importantly, the incorporation of nanocarriers did not negatively impact critical properties of the bone cement, including curing time, water absorption, compressive and flexural strength and fracture toughness. Additionally, cytocompatibility assays confirmed that the system supported osteoblast viability and mineralization, indicating its suitability for clinical applications (Figure 15B and C).

#### Polylactic acid

Although PMMA has been extensively utilized in orthopedic clinics as a drug delivery matrix, its long-term persistence *in vivo* raises concerns regarding bioaccumulation and immune reactions. Consequently, recent research has shifted toward developing drug delivery platforms based on biodegradable materials, which obviate the need for secondary surgical removal as they undergo complete resorption in the body. This not only lowers healthcare costs but also enhances patient compliance. Polylactic acid (PLA), a biodegradable thermoplastic synthesized via polycondensation of lactic acid monomers, possesses ester linkages that undergo hydrolytic degradation to release lactic acid—an endogenous metabolite safely processed by human body [262]. Owing to its favorable mechanical properties and biocompatibility, PLA has been broadly applied in medical devices such as fixation anchors, rods, plates, screws, pins, meshes and drug delivery systems [263]. Notably, the mechanical characteristics of PLA can be finely tuned by adjusting parameters such as molecular weight, degree of polymerization and crystallinity, while its degradation rate can be modulated through molecular structure design [264]. Thus, features render PLA highly processable and adaptable to advanced manufacturing techniques, enabling its application in the treatment of complex bone defects.

**Figure 16:**
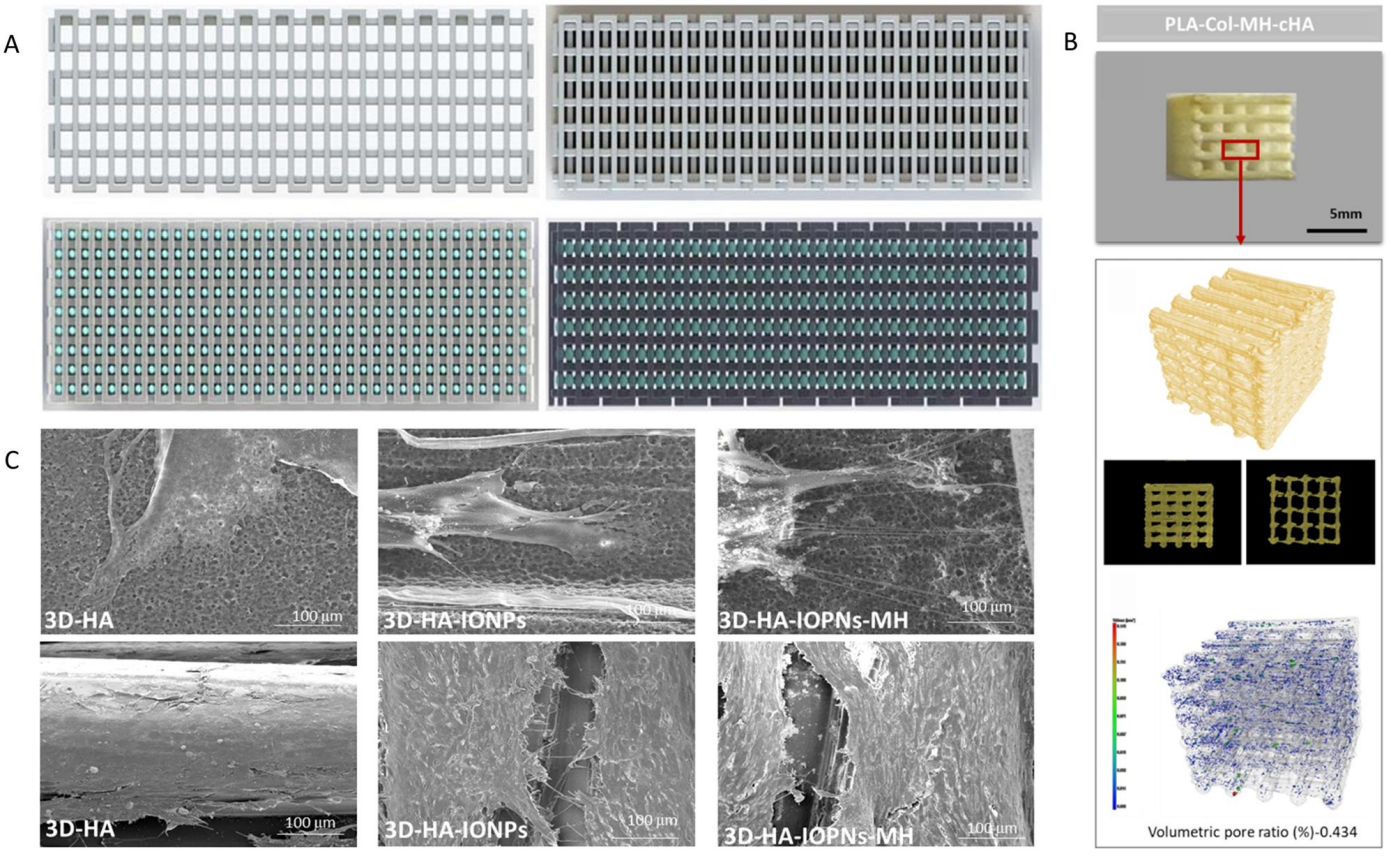
Design of 3D-printed PLA scaffolds. (**A**) Schematic diagram of different 3D scaffolds designed using computer-aided modeling [265]. Copyright 2023, Royal Society of Chemistry. (**B**) Image of the 3D PLA-Col-MH-cHA scaffold. (**C**) Representative SEM images of hBMSCs cultured on the surface of the PLA 3D scaffold for 5 days (top) and 15 days (bottom). Reproduced with permission from Ref. [266], Copyright 2019, Elsevier.

Gao *et al*. [265] employed 3D printing technology to fabricate PLA/nHA scaffolds with vertically orthogonal and staggered orthogonal configurations. A vancomycin-loaded chitosan (CS) hydrogel was applied to the scaffolds to establish a local antibiotic delivery platform. The resulting constructs exhibited high porosity and a well-connected three-dimensional network, improving surface hydrophilicity and promoting osteoblast adhesion and proliferation. *In vitro* release assays confirmed sustained-vancomycin release for over eight weeks and demonstrated effective inhibition of *S. aureus* growth (Figure 16A). Martin *et al*. [266] and colleagues developed an alternative approach using fused deposition modeling (FDM) with 1.75 mm PLA filaments to print scaffolds, followed by surface modification via alkaline hydrolysis to increase hydrophilicity and roughness, thereby enhancing cellular attachment. The scaffolds were further functionalized with collagen and nanohydroxyapatite, producing a composite structure (PLA–Col–MH–cHA) that featured a highly organized porous architecture and mechanical properties approximating those of cancellous bone. This design supported cell infiltration and angiogenesis. The local sustained release of minocycline provided robust antibacterial and antibiofilm effects, effectively preventing IAI (Figure 16B and C). In another study, these scaffolds were loaded with iron oxide nanoparticles (IONPs) synthesized via a simple aqueous co-precipitation method at ambient temperature. The resulting 3D-printed constructs exhibited strong bioactivity, osteoinductive capacity, compatibility with primary osteoblasts and antimicrobial activity against *S. aureus* [267]. Furthermore, Martin’s group demonstrated that the fabricated scaffolds maintained high compressive strength, favorable wettability and a uniform macroporous structure analogous to natural bone.

Shakeri *et al*. [268] developed a trilayered fibrous membrane composed of gelatin (GT) and PLA to provide temporally controlled, spatially targeted drug release. The outer GT layer, oriented toward soft tissue, was loaded with thymol (THY) for rapid antibacterial action. The inner PLA layer, facing the bone defect, incorporated simvastatin (SIM) for sustained-osteogenic stimulation. The middle layer, comprising mixed GT/THY and PLA/SIM fibers, reinforced interlayer cohesion and enabled functional transition across the interface. This stratified design achieved a staged release profile, with THY released rapidly to inhibit early biofilm formation, while SIM was gradually released over 35 days to promote osteogenic differentiation and angiogenesis, all within biologically safe concentration ranges. By replicating the physiological architecture and functional gradients of natural periosteum, this multilayer system delivered localized, differential biological regulation at the soft–hard tissue interface. The approach presents a novel strategy for the development of biomaterials with integrated anti-infective and osteogenic properties.

### Nanomaterials

Nanomaterials are engineered substances with dimensions ranging from 1 to 100 nanometers in at least one dimension. Their physical and chemical properties (e.g. specific surface area, quantum effects and surface energy) differ significantly from those of macroscopic materials. Their functions can be precisely customized through size control, surface modification and multilevel structural design [269]. The surface properties of nanomaterials are similar to those of natural bone, and they effectively promote the adhesion, proliferation and differentiation of bone cells [270]. The high specific surface area and functionalizable surface area of nanomaterials make them ideal drug carriers, facilitating targeted delivery and sustained release of antibiotics while providing multiple anti-infection mechanisms. Moreover, nanostructuring significantly increases the strength and toughness of bone repair materials through precise control of porosity and structural properties, providing an ideal microenvironment for angiogenesis and tissue regeneration [271]. Nanomaterials are primarily classified into two categories on the basis of their chemical composition: organic nanomaterials and inorganic nanomaterials. Organic nanomaterials are composed of organic compounds derived from natural products or obtained through chemical synthesis. Common forms include lipid-based, polymer-based and protein- and nucleic acid-based nanomaterials. These materials exhibit excellent biocompatibility and structural tunability [272]. In comparison, inorganic nanomaterials do not contain C-H bonds and possess greater chemical stability and mechanical strength. Common types of materials include metals, ceramics, magnetic materials, quantum dots, carbon-based materials and silicon dioxide. Owing to their high stability, good surface adjustability and diverse functions, inorganic nanomaterials are widely used in fields, such as bioimaging, antibacterial applications and bone repair [273]. In the various biocomposite systems discussed above, nanomaterials are often introduced in the form of functional enhancers to improve antibacterial properties, impart controlled release properties or enhance osteogenic activity. However, with the development of nanotechnology, nanomaterials are not only auxiliary factors but are also gradually becoming the core platform for building multifunctional integrated treatment systems. This section focuses on the key role of nanomaterials in systemic treatment design and explores their unique advantages in terms of infection control, bone regeneration and smart regulation.

#### Function integration characteristics: building a multifunctional integrated platform

**Figure 17:**
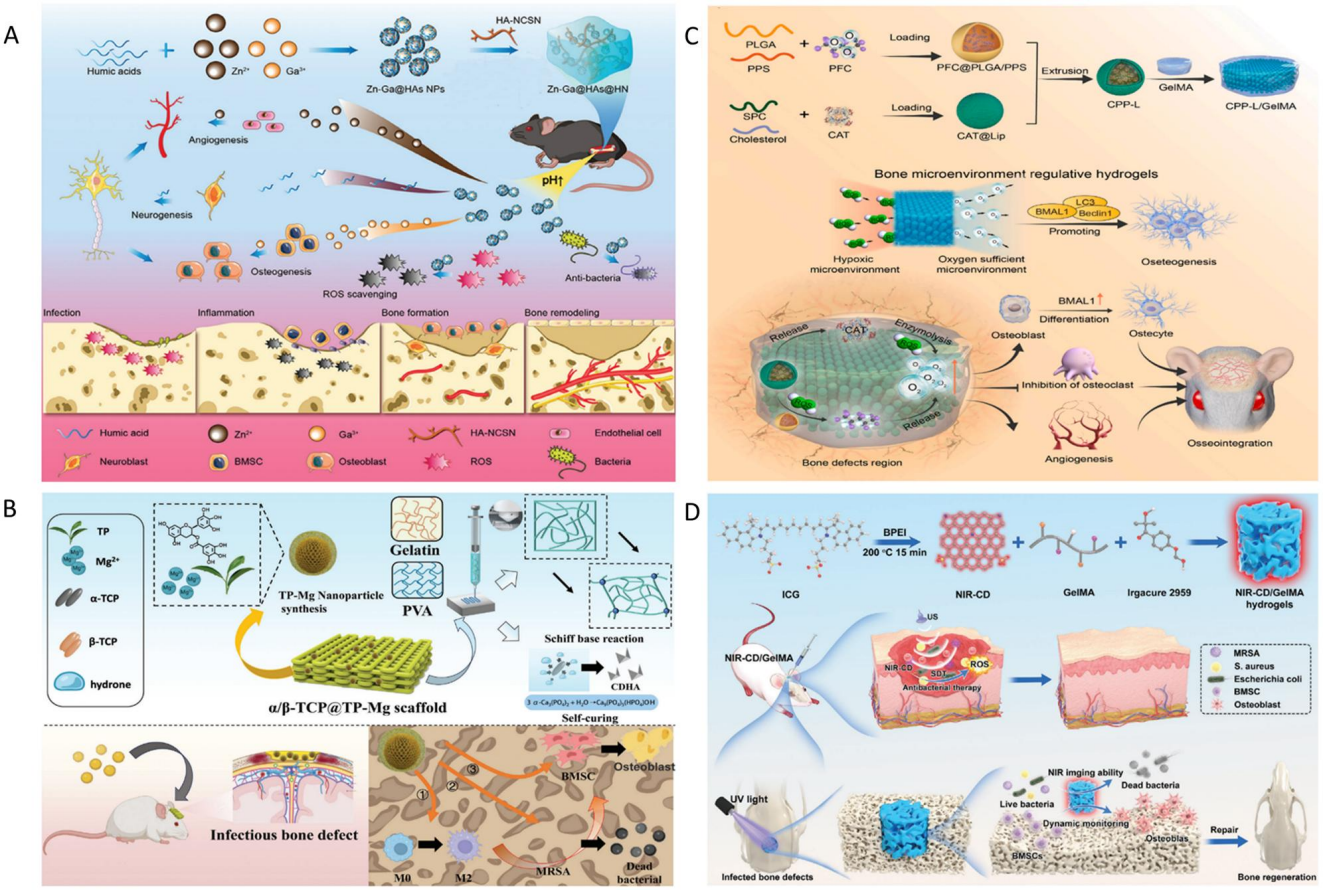
Schematic representation of the design and applications of multifunctional nanomaterials. (**A**) Schematic illustration of the application of the Zn-Ga@HAs@HN hydrogel in promoting bacteria-infected bone fracture healing. Reproduced with permission from Ref. [275], Copyright 2024, Wiley. (**B**) Schematic diagram of self-setting bioactive bone repair scaffold loaded with TP-Mg for the treatment of infectious bone defects. Reproduced with permission from Ref. [276], Copyright 2024, Wiley. (**C**) Schematic illustration of bone microenvironment regulative hydrogels with ROS scavenging and prolonged oxygen-generating for enhancing bone repair. Reproduced with permission from Ref. [279], Copyright 2023, Elsevier. (**D**) Schematic illustration of the preparation procedure of the NIR-CD/GelMA and its application for repairing IBD. Reproduced with permission from Ref. [282], Copyright 2024, Wiley.

Nanomaterials can form stable composite interfaces with different types of matrix materials (e.g. ceramics, metals and hydrogels), significantly improving the structural integration and biocompatibility between materials. For instance, Wu *et al*. [274] modified the surface of metal scaffolds with a nanohydroxyapatite coating, which not only increased the interface affinity between the implant and bone tissue but also provided a functional platform for further loading of antimicrobial nanoparticles or drug delivery systems. In addition, introducing nanoparticles into hydrogel or polymer microsphere systems can also enhance their mechanical properties, antibacterial capabilities and controllability of drug release, overcoming the limitations of traditional hydrogels, which are poor in resilience and single function. Zha *et al*. [275] developed a nanocomposite hydrogel containing zinc-gallium-humic acid nanoparticles, which exhibited powerful antibacterial effects and promoted angiogenesis, osteogenesis and nerve healing through the timely release of Zn^2+^, Ga^3+^ and humic acid (Figure 17A).

Nanomaterials are also often used as ‘functional carriers’ or ‘multifunctional modules’ in composite systems, achieving organic integration of structural levels and therapeutic mechanisms. For illustration, embedding functional nanoparticles inside 3D-printed scaffolds can create functional regions with gradient distributions, enabling spatiotemporal coordination and control from structural support to drug release (Figure 17B) [276]. Certain multifunctional nanosystems (e.g. metal-organic frameworks, MOFs) can perform three tasks simultaneously, namely, antibacterial, osteogenic and drug delivery, greatly simplifying the complexity of material design [277].

The highly engineering-friendly properties of nanomaterials enable them to serve as a ‘universal integration platform,’ facilitating the flexible incorporation of functional modules across various material systems and promoting cross-type integration and functional synergy between materials. This not only expands the design space for biocomposites but also provides technical support for multifunctional integrated treatment strategies, driving the transition from ‘multimaterial combinations’ to ‘systemic treatment platforms’.

#### Intelligent response mechanism: constructing a microenvironment release system

In previous studies, classic delivery materials such as hydrogels and microspheres have been widely used for the local release of drugs in IBD because of their good biocompatibility and drug loading capacity. However, such materials are mostly passive release systems, with drug release rates typically controlled by diffusion or material degradation rates. They lack the ability to sense changes in the tumor microenvironment and may struggle to achieve precise control in complex, dynamic infection environments. Therefore, in recent years, researchers have attempted to expand drug delivery systems toward environmental responsiveness to enhance the targeting and safety of treatment.

The core mechanism of the responsive release system lies in identifying pathological characteristics specific to the infected site, for example, acidic pH, elevated ROS levels and increased expression of specific bacterial enzymes (e.g. phospholipases and metalloproteinases) [278]. Nanomaterials can respond to these stimuli through structural design, thereby triggering the degradation, expansion or opening of pores in drug carriers to achieve the targeted release of therapeutic agents. Sun *et al*. [279] designed a novel responsive release system, CPP-L/GelMA hydrogel, whose core mechanism is to precisely regulate the microenvironment of bone defects in response to excessive ROS and hypoxic conditions. Hydrogels loaded with the antioxidant enzyme catalase (CAT) and ROS-responsive oxygen-releasing nanoparticles (PFC@PLGA/PPS) can decompose hydrogen peroxide into oxygen in low-oxygen environments and intelligently trigger the continuous release of oxygen in response to changes in ROS levels. This system not only effectively alleviates oxidative stress caused by hypoxia, but also promotes bone formation and angiogenesis while inhibiting osteoclast activity. Through the Nrf2-BMAL1-autophagy pathway, CPP-L/GelMA hydrogel significantly enhances the differentiation potential of osteoblasts, providing an innovative multifunctional strategy for the repair of IBD (Figure 17C).

In recent years, MOFs, hollow nanospheres, liposomes and other materials have been widely used to construct such smart response carriers and have demonstrated good therapeutic effects *in vivo* infection models [280]. For example, Sun *et al*. [281] developed a pH-responsive Cu-modified hollow manganese dioxide nanoparticle-loaded PTH-directed gelatin scaffold. This scaffold uses an acid-sensitive bond to encapsulate functional structures. In the acidic environment of the infected area, MnO_2_ decomposition accelerates the release of PTH/Cu^2+^, thereby concentrating the attack on pathogens while minimizing the impact on normal tissue. ROS-responsive nanosystems utilize high-ROS environments to induce structural breakdown of the material, thereby releasing the encapsulated drug. These systems can be used in combination with anti-inflammatory factors and bone growth factors for the dual regulation of infection control and tissue repair.

In recent years, a number of new smart response release material designs have emerged. Tang *et al*. [282] synthesized NIR carbon dots (NIR-CDs) with enhanced sonodynamic antibacterial properties, narrow bandgap NIR imaging ability and negative charge-enhanced osteogenic activity. They reported that this material generates a large amount of ROS under ultrasound irradiation, effectively inhibiting the growth of methicillin-resistant MRSA and promoting bone defect repair by stimulating the osteogenic differentiation of BMSCs (Figure 17D). In the future, responsive systems are expected to be integrated with sensor technology and visual diagnostic and therapeutic methods, further promoting the development of personalized and intelligent treatments for IBD.

## Preclinical *in vivo* research progress on the treatment of IBD with biocomposites

With respect to the development of biocomposites, although *in vitro* studies allow us to understand and evaluate the effects and safety of biomaterials at the cellular level, the theoretical advantages and preliminary experimental results do not ensure their safety and efficacy in the human body. Considering ethical, safety, economic and regulatory issues, bone implants should undergo preclinical *in vivo* tests to ensure that they function effectively and safely in humans [283]. This is also a key factor in translational research. Researchers can further investigate the antibacterial activity and osteogenic ability of biocomposites in complex physiological environments to optimize the material design and improve their performance.

### Selection and design of *in vivo* models

The close evolutionary relationships and anatomical similarities between species suggest that they have the same molecular background, biochemical mechanisms and similar physiological functions. Therefore, the more similar an animal model is to humans, the more suitable it is for research purposes. Many animal models, such as mice, rabbits, dogs, sheep and pigs, have been developed to simulate the physiological environment of the human body. To simulate various types of IBD, many defect sites (e.g. femur, skull, ulna) and pathogenic bacteria types (e.g. *S. aureus*, MRSA and *E. coli*) have been explored [284] (Table 2). When a specific animal species is selected as an experimental model, multiple factors must be considered. First, the selected animal model should have significant physiological and pathophysiological similarities to humans. Second, it must be possible to operate and observe multiple subjects within a short period [285]. Most importantly, IBD animal models need to consider the animal immune response, the course of infection and pathological reactions. That is, they must not only withstand mechanical loads and the challenges of bone regeneration but also be able to simulate chronic inflammation and persistent infection. Other selection criteria include low cost of acquisition and care, easy availability of animals, social acceptance, tolerance to captivity and ease of husbandry [286].

**Table 2:** Examples of preclinical studies on antibacterial biocomposites.

Materials	Animals	Model	Postoperative endpoint	Inoculation methods	Antibacterial agents	References
HA/Col scaffold	Rat	3 mm drill hole in lateral femoral condyle	1 week	Injection of 1 × 10^7^ CFU *S. aureus* into femoral defect	Cefotiam and Vancomycin	[301]
PU/Ag-THA scaffold HA/PU scaffold	Rat	Ø: 3.5 mm × 5 mm the lateral condyle of the femur	10 days	Inoculation of 1 × 10^6^ CFU/mL *S. aureus* into bone defect	silver nitrate (AgNO3) and Tannin (T)	[119]
ABVF-BG	Rat	4.2 mm hole the tibial metaphysis	At the same time as inoculation	Drill hole at femoral condyle and inject 1 × 10^8^ CFU/10 µL *S. aureus*	Vancomycin	[302]
Stainless steel plates	New Zealand white rabbit	Mid-diaphyseal osteotomy in femur using saw blade	4, 6 weeks	Implants preincubation with 1 × 10^6^ CFU/mL MRSA for 12 h	Perfluoropolyether lubricant	[303]
Stainless steel plates	Dorsetcross ewes	Mid-diaphyseal osteotomy in unilateral tibia using oscillating saw	4, 12 weeks	Inoculation in fracture site, 2.5 mL, 1 × 10^6^ CFU/mL	N, N-dodecyl, methyl-PEI	[304]
Titanium rods	New Zealand white rabbit	2 defects on the femur (*d* = 2 mm)	7, 14, 60 days	Implants preinoculation (1 × 10^8^ CFU/mL, 15 μL)	Fusion peptide	[305]
Titanium K-wires	New Zealand white rabbit	Mid-diaphyseal osteotomy in tibia using a micro saw	4 weeks	Injection into the medullary cavity, 100 μL, 1 × 10^5^ CFU/mL	Cu^2+^	[306]
PCL scaffold	New Zealand white rabbit	Cylindrical defect in tibia	8 weeks	Injection into the marrow cavity, 100 μL, 1 × 10^5^ CFU/mL	Ag^+^	[307]
PEEK implants	Rat	Cylinder defect on the outside of the vertical femur (*d* = 2 mm, *h* = 5 mm)	4 weeks	Injection into femoral cavity, 30 μL, 1 × 10^4^ CFU/mL	GS	[308]

Small animal models are commonly used to verify concepts and perform preliminary tests on the degradation and biocompatibility of biomaterials. These models offer advantages, such as low cost, ease of operation and short experimental duration [287]. For instance, many researchers have used rat skull and long bone defect models in the preliminary evaluation of the anti-infection effects and bone regeneration capacity of materials. However, although these models are advantageous for early screening, their bone tissue structure, stress environment and healing ability differ significantly from those of humans [288]. Therefore, the research results need to be extrapolated with caution. In contrast, large animal models, such as dogs, sheep or pigs, are important models in preclinical research because their bone structure is similar to that of humans in terms of stress load, bone density and bone healing mechanisms [289]. For example, dog tibia and sheep radius defect models are more closely aligned with human bone biology. These models have higher reference values when evaluating the mechanical properties and degradation rates of materials under simulated load-bearing conditions [290]. However, large animal experiments are more expensive, require longer experimental durations and involve more complex ethical issues. Therefore, these methods are generally performed for further functional studies after the preliminary validation of materials. To summarize, no single animal model is suitable for all purposes in a research field; Therefore, multiple model systems are usually needed to establish a comprehensive understanding [291].

When *in vivo* IBD models are designed, various factors need to be considered, including the joint size, bone thickness, depth and diameter of the defect, skeletal maturity age, joint load distribution and burden and ethical concerns related to animal handling [285]. In addition, unlike ordinary bone defect models, IBD models require local inoculation with pathogenic bacteria to simulate the infectious microenvironment. There are three main routes of administration: intraosseous administration, local administration via the nutrient artery and systemic administration via intravenous injection [292]. To reduce costs and animal suffering, researchers often use critical bone defects as bone defect experimental models. A critical bone defect is defined as the smallest defect size that cannot be repaired without external intervention [293]. This model simulates clinical conditions and assesses the bone regeneration ability of biomaterials. The critical bone defect size varies between models depending on the species and skeletal maturity. Combined models of bone defects and exogenous infections are effective platforms for the *in vivo* evaluation of the osteogenic and anti-infection properties of materials. Segmental defects are created by drilling in the femur or tibia, with hole diameters of 0.8–3.5 mm, depending on the animal used. In addition to segmental defects, rod-shaped implants are also commonly placed in the femur or tibia medullary cavity to evaluate the osteogenic performance of materials [294]. This method can be further refined to construct a bone marrow infection model to study the osteogenic and anti-infection properties of materials simultaneously [295].

### Preclinical *in vivo* evaluation of material performance

During the development of biocomposites, *in vivo* evaluation of various material properties is a key step in determining whether these materials can be used in clinical applications. *In vivo* experiments provide a multidimensional test of materials in dynamic, load-bearing and pathological environments, validating their functional performance in complex biological settings. When conducting *in vivo* evaluations of biocomposites, researchers should focus on the comprehensive performance of the material in terms of both antibacterial and osteogenic properties, taking into account the characteristics of the IBD model. The materials used in these experiments should have effective antibacterial properties, be able to inhibit the proliferation of bacteria in bone defects, and prevent excessive inflammatory responses during bone repair. For instance, by implanting BG/polylactic acid composite scaffolds in infection models, it is necessary not only to observe whether they promote new bone formation within 6–12 weeks but also to monitor bacterial colonization and inflammatory cell infiltration to verify the anti-inflammatory effects of the material [296]. In addition to assessing short-term antibacterial and bone repair effects, long-term effects, which typically require long-term experiments lasting six months or more, are equally important [297].

The repair process of IBD is a multistage, dynamic process. Different biocomposites have been evaluated *in vivo* in animal experiments for the repair of IBD in different ways, mainly in terms of the focus of evaluation and the choice of methods. For materials with antibacterial properties, for example, metal ion or antibiotic carrier composites, experimental evaluations typically focus on bacterial culture and antibacterial activity testing, assessing the antibacterial effectiveness of the material through regular bacterial colonization testing and colony-forming unit (CFU) counts [298]. In addition, since infection triggers a local immune response, it is also necessary to focus on inflammatory cell infiltration, proinflammatory cytokine expression and immune regulatory functions when evaluating the performance of materials in IBD models. These factors may have a more significant impact on bone healing than bone defects alone [298]. For materials with a primary osteogenic effect, for example, bioceramics or calcium phosphate composites, evaluation focuses more on bone density, new bone formation and changes in bone structure. Commonly used evaluation methods include micro-CT scanning and histological section analysis [299]. The mechanical properties of the material also need to be verified through compression testing and biomechanical evaluation to ensure that it can provide sufficient support during the repair process [300]. Overall, different types of biocomposites are evaluated via different methods depending on their characteristics, but all methods need to comprehensively consider factors such as antibacterial properties, bone repair effects and biocompatibility.

## Regulatory pathways for biocomposites conversion

Regulatory science is an emerging interdisciplinary field centered around the management of the entire lifecycle of medical products. It focuses on the development of new tools, standards and methods to assess the safety, efficacy, quality and performance of these products systematically [309, 310]. Its core mission is to provide scientific evidence for regulatory decisions, balance innovation and risk and ultimately serve public health. Unlike traditional scientific research, which is ‘curiosity-driven’, regulatory science is distinctly ‘mission-oriented’, concentrating on addressing scientific challenges in regulatory practice [311].

**Figure 18:**
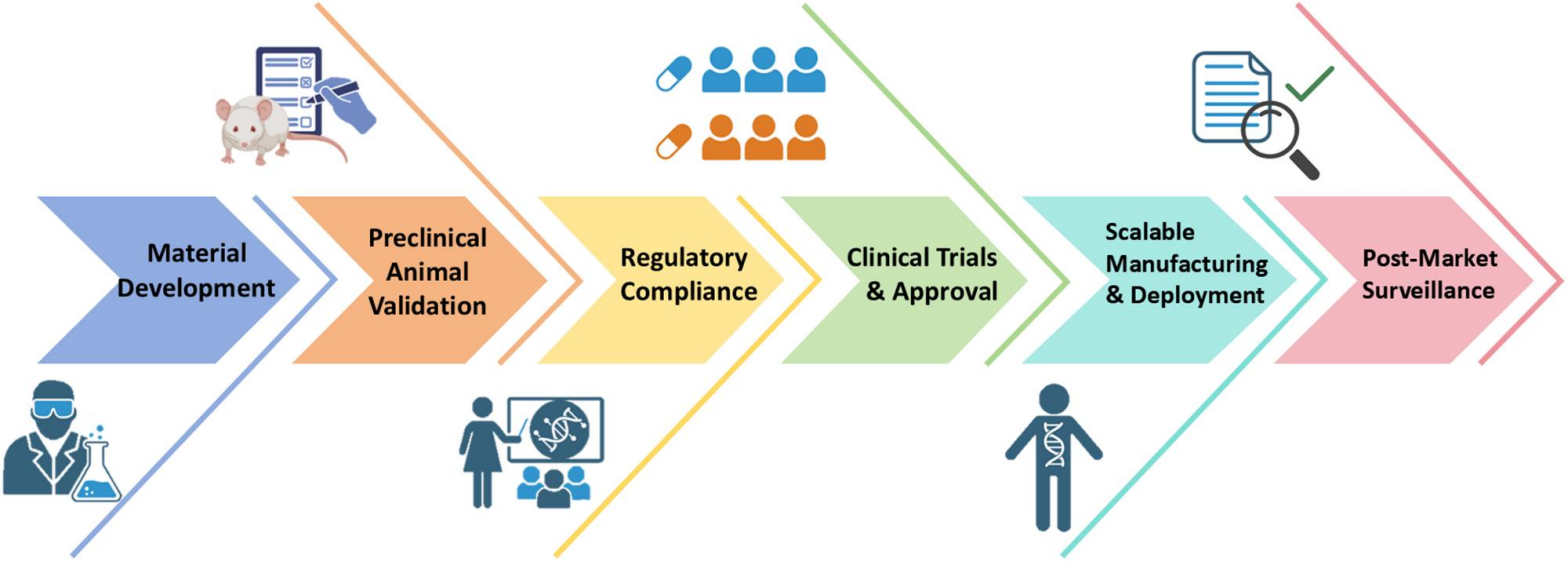
The process of transforming biomaterials from design to medical products.

Biomaterials have been widely used in fields, such as medical devices and tissue engineering. Before these materials become official medical products, they must undergo a series of scientific evaluations and regulatory processes (Figure 18; Table 3). Traditional regulatory frameworks often focus on assessing single-function materials, whereas biocomposites, owing to their multifunctional complexity, cannot be fully evaluated by traditional standards. In this context, regulatory science must shift its focus from safety and efficacy to a full lifecycle assessment. This process should encompass not only the regulation of raw material sources, production processes and preclinical trials for biocomposites but also long-term monitoring and postmarket surveillance in clinical applications [311]. Therefore, biocomposite regulation must integrate new technologies, such as 3D printing and nanotechnology to achieve more efficient multidimensional monitoring and data feedback mechanisms.

**Table 3:** Clinically applied biocomposites.

Material	Implant site	Function	Application	Patient characteristics	Outcome	References
PerOssal (HA + CaSO_4_)	Distal tibia	1. Local antibiotic delivery 2. Biodegradable 3. Osteoconductive	Fill bone defect region and deliver antibiotics locally to control infection.	Chronic osteomyelitis (Cierny-Mader type 3A) after distal tibial open fracture	AP and lateral X-rays at 6-and 9-month post-op showed good bone reconstruction	[318]
S53P4 (bioactive glass)	Tibia	1. Antibacterial 2. Osteoinductive 3. Angiogenic	Fill cavitary bone defects in chronic osteomyelitis and assist in local infection treatment without additional local antibiotics.	Post-traumatic tibial osteomyelitis	Two-year follow-up: patient asymptomatic, no active draining sinus; plain radiographs still showed presence of bioactive glass	[319]
Antibiotic-impregnated calcium phosphate cement	Tibia	1. Local antibiotic delivery 2. Biodegradable 3. Osteoconductive	Applied in treatment of *S. aureus* infection following open-wedge high tibial osteotomy, achieving local antibiotic release and bone healing while avoiding correction loss and revision surgery.	Post-open-wedge high tibial osteotomy with *S. aureus* infection	At 8 weeks post-op: restored knee ROM; 12 weeks: good bone healing; 3-year follow-up: no loss of correction	[320]
antibiotic cement coated interlocking intramedullary nails（ACC-IMN）	Midshaft tibia	1. Antibacterial 2. Provides stable support	Through a single-step insertion of ACC-IMN, the fracture site was rapidly stabilized, while sustained local antibiotic release further controlled infection.	Septic tibial nonunion	Long-term follow-up: AP and lateral X-rays showed complete tibial healing with ACC-IMN in place	[321]
Cerament-V	Ankle	1. Provide stable support 2. Biodegradable 3. Antibacterial 4. Osteoconductive	Intramedullary nail coated with Cerament-V, secured with locking screws for stability.	Diabetic patient with neuropathy and retinopathy; fifth metatarsal fracture complicated by Charcot neuroarthropathy progressing to osteomyelitis and ulcer	Follow-up: ankle and subtalar fusion, good bone healing and effective infection control	[322]
PLGA nanoparticles	Periodontal pocket	1. Local antibiotic delivery 2. Immunomodulatory	Coated in periodontal pocket post-treatment with 20% doxycycline-loaded PLGA nanoparticles for sustained infection control.	Type 2 diabetes with chronic periodontitis	Pocket depth reduced, periodontal pathogen levels decreased; 3-month post-treatment: significant HbA1c reduction	[323]

The ultimate goal of the translation of biocomposites is to develop safe and effective medical products that benefit human healthcare [312]. In the basic research phase, the core of regulatory science lies in establishing a dual framework that supports both innovation and standardization. The physicochemical properties, biocompatibility and safety of biocomposites must be tested. All tests must adhere to relevant standards and methods to ensure compliance throughout the development process [309]. The regulation of emerging technologies, for example, nanotechnology, must incorporate risk prediction mechanisms at the design stage of biocomposites. The unique structure of nanomaterials can bypass traditional absorption pathways and methods, leading to potential new properties in terms of physicochemical characteristics, pharmacology, toxicology and pharmacokinetics. For instance, when developing nanohydroxyapatite bone repair materials, it is necessary to establish computational toxicology models on the basis of their particle size distribution and surface modifications to predict the risks of liver and kidney function damage under long-term exposure. For illustration, the National Medical Products Administration (NMPA) of China released the ‘Guidelines for Safety Evaluation of Medical Devices Using Nanomaterials’. These guidelines predict the *in vivo* behavior of nanomaterials through precise characterization of physicochemical parameters, such as surface charge and particle size distribution [312]. For novel bioactive biocomposites, regulatory agencies can promote the establishment of standardized material databases, integrating data on their physicochemical properties, degradation behavior and cellular responses. This would provide a comparative benchmark for subsequent research.

In the preclinical development phase, regulatory focus should be on validating material performance and adaptability to specific application scenarios. Regulatory agencies need to establish a phased review mechanism: first, mandatory testing of the material’s mechanical properties, degradation kinetics and compliance with the ISO 10993 series standards; second, for specific applications, for example, orthopedic implants or drug delivery systems, a differentiated evaluation system must be developed [313]. For example, for orthopedic implants containing antimicrobial components, the ‘Guidelines for Registration and Review of Antimicrobial Performance of Orthopedic Implants’ clearly require preclinical studies to include key indicators, such as coating physicochemical properties, antimicrobial substance release kinetics and *in vitro* antimicrobial efficacy to verify their safety and effectiveness [311]. For 3D printing technology, the regulatory focus should be on standardizing the process-performance relationship during the preclinical development phase. By utilizing multiphysics simulations and machine learning to predict *in vivo* performance, reliance on animal testing can be reduced while improving predictive accuracy. For personalized orthopedic implants, for instance, it is necessary to define the quantitative correlation rules between key process parameters and critical quality attributes based on the ASTM F3301 additive manufacturing standard. Additionally, a comprehensive traceability system covering the entire ‘design-print-postprocessing’ chain should be established [314, 315].

In the clinical trial phase and postmarket regulation, the task of regulatory science is to ensure data authenticity and patient safety. For multicenter clinical trials of biocomposites, a dynamic risk assessment mechanism must be established, with a focus on the long-term performance of the materials in complex physiological environments. For example, the EU Medical Device Regulation (MDR) requires companies to submit risk-benefit analysis reports on the basis of actual clinical data and clearly define the postoperative follow-up period [316]. The dynamic safety and functional efficacy of biocomposites must be continuously validated through a multidimensional postmarket surveillance system. The core of regulatory science in this context lies in constructing a dynamic risk assessment model based on real-world data (RWD) to address potential risks arising from the interaction between the material and the host microenvironment after long-term implantation. For instance, by integrating multimodal data from electronic health records (EHRs), patient registry databases and feedback from smart implants, real-time monitoring of material mechanical property degradation or biocompatibility shifts can be achieved, along with the establishment of predictive failure thresholds. This strategy not only compensates for the spatial and temporal limitations of traditional clinical trials but also facilitates a regulatory paradigm shift from a ‘passive response’ to an ‘active early warning’ through AI-driven anomaly signal mining technology. For illustration, the EU MDR requires companies to implement at least 10 years of postoperative follow-up for orthopedic implants containing degradable components combined with machine learning to analyze the material degradation rate [317]. Moreover, regulatory science in this phase can balance innovation and risk control. For instance, the FDA’s ‘Breakthrough Device Program’ provides an accelerated review pathway for breakthrough combination products, allowing companies to submit process validation data in phases, thereby shortening the development cycle [317].

## Challenges and prospects

Although significant progress has been made in recent years in the development of biocomposites for infection control and bone regeneration, their clinical translation still faces multiple challenges. These challenges do not arise solely from insufficient material performance, but are more closely associated with the complexity of the clinical pathological environment, the *in vivo* realization of functional synergy and the gap between current evaluation systems and clinical decision-making.

### The gap between real pathological conditions and material design assumptions

Most current biocomposites are designed and validated under relatively idealized experimental conditions. However, clinical IBD is typically characterized by complex pathological features, including mature biofilms, persistent inflammatory responses and compromised local blood supply. Under such circumstances, the antibacterial or osteogenic performance demonstrated *in vitro* or in simplified animal models is often partially attenuated or even negated *in vivo*. Therefore, future research should place greater emphasis on incorporating real clinical pathological conditions into the early stages of material design and validation. For example, mature biofilm models, chronic inflammatory states and compromised bone bed environments should be systematically integrated into functional evaluations of materials, thereby improving the clinical relevance and translational value of experimental findings.

### Lack of clear design logic for synergistic infection control and bone regeneration

In the treatment of IBD, infection control and bone regeneration do not occur as parallel processes but rather exhibit distinct stages and condition dependencies. However, current multifunctional biocomposites primarily adopt a design approach based on functional addition, without adequately considering the differences in the temporal scale and microenvironmental requirements of each function. When infection is not effectively controlled or when the inflammatory load is high, osteogenic signals are often unable to exert their effects. Conversely, persistent or excessive antibacterial stimulation may inhibit bone regeneration. Therefore, future material design must shift from a ‘functional coexistence’ model to one focused on ‘functional sequence and temporal window regulation’. By employing rational temporal release or spatial allocation strategies, infection control functions can create stable time and space conditions for subsequent vascularization and bone regeneration, thereby achieving true functional synergy.

### Clinical translation is limited by the mismatch between evaluation systems and clinical decision-making needs

A key challenge in the clinical translation of biocomposites is that the evaluation metrics used in experimental studies are insufficient to support clinical decision-making. Current research often focuses on short-term antibacterial effects or osteogenic markers, with limited attention to clinical core issues such as the risk of infection recurrence, long-term osseointegration stability and functional recovery. In clinical practice, the ability of a material to reduce recurrence rates, shorten treatment cycles and support single-stage repair is often of greater value than improvements in function at a single time point. Therefore, future research needs to establish a more comprehensive evaluation system that closely aligns with clinical needs. This system should systematically assess antibacterial efficacy, bone regeneration quality and safety within the same model, thus, enhancing the clinical interpretability of experimental results.

### Challenges in scaling up production and building quality control systems for biocomposites

Many multifunctional composites rely on complex laboratory processes that achieve optimal performance under small-scale conditions. However, when transitioning to clinical applications, they face dual bottlenecks related to manufacturability and verifiability. The longer the process chain, the greater the batch-to-batch variability; the more complex the composition, the more difficult quality control becomes. In particular, the synergistic system of antimicrobial components and bioactive factors is highly sensitive to content, release profiles and spatial distribution. Even slight deviations can lead to inadequate antibacterial performance or cytotoxicity. Therefore, the core challenge of clinical translation is not merely scaling up production but establishing reproducible, traceable and GMP-compliant process windows and key quality attributes. During the design phase, it is essential to consider which structures and components are indispensable and which can be sacrificed, trading off engineering controllability for clinical reproducibility and regulatory acceptability.

### Potential breakthroughs in future research

Traditionally, synergy has often been understood as the coexistence of antibacterial and osteogenic functions within a single material. However, in the complex infection microenvironment, this static combination frequently faces issues of functional conflict and mismatched therapeutic windows. Looking ahead, the development of biocomposites for treating IBD is expected to achieve breakthroughs in several key areas.

First, material design concepts will gradually shift from enhancing single functions to regulating the entire therapeutic process. Through temporal and interfacial functional design, the goal will be to guide a smooth transition from infection clearance to tissue regeneration. Second, antibacterial strategies will increasingly focus on long-term relapse prevention, rather than just short-term bactericidal effects, to address the clinical reality of IBD’s high recurrence rate. Mature biofilms not only serve as physical barriers but also form stable microecological structures and immunosuppressive environments, which are the core pathological basis of IBD’s high recurrence rate. Therefore, relying solely on high concentrations of antibiotics or metal ion release is often insufficient to achieve long-term control while maintaining tissue compatibility. Future breakthroughs will shift from enhancing bactericidal capabilities to reconstructing the infection niche. For example, the recently proposed ‘bacteria-fooling bacteria’ strategy offers a new approach for biofilm intervention [324]. This method uses engineered inactivated homologous bacteria as biomimetic carriers to actively embed in mature biofilms and deliver drugs within the membrane, leveraging the natural recognition and adhesion properties between pathogens.

Finally, with the further alignment of material research with clinical needs, more clinically relevant models and evaluation frameworks will become key drivers for clinical translation. Therefore, ‘synergy’ in the future should be understood as a cross-scale, cross-phase and cross-mechanism system engineering. It requires the formation of a closed-loop between materials science, immunology, microbiology and clinical needs. It also necessitates the establishment of a quantifiable balance model between efficacy and safety and the clarification of translatable paths within the framework of individualized manufacturing and regulatory science. Only when biocomposites can establish a predictable, controllable and sustainable dynamic synergy between infection control and tissue regeneration will the treatment of IBD progress from passive repair to active reconstruction.

In conclusion, the application prospects of biocomposites in IBD treatment remain broad. However, their further development relies on a deeper understanding of clinical challenges and systematic exploration of the mechanisms underlying functional synergy *in vivo*. By establishing closer connections between material design logic, evaluation systems and clinical orientation, future research is expected to narrow the gap between experimental studies and clinical applications, driving the field toward higher levels of advancement.

## Conclusion

This article discusses the latest research findings on multiple biocomposites for treating IBDs, highlighting their multifunctionality and integrated therapeutic strategies. This study provides a detailed analysis of the synergistic effects of biocomposites with different compositions on antibacterial properties, osteogenesis and degradability. This study also provides a theoretical foundation for the development of new bone graft materials that combine both antibacterial and osteogenic functions for clinical use. Traditional methods for treating IBDs mainly rely on antibiotic treatment and multistep surgical interventions, including infection focus clearance and necrotic tissue removal. However, these methods have significant limitations. Long-term antibiotic use may give rise to antibiotic-resistant bacteria, reducing the effectiveness of treatment and causing secondary infections. Surgical treatment is complex and invasive and often involves multiple surgeries, which increase the risk of infection and medical costs.

Biocomposites, which integrate osteogenesis-promoting and antibacterial components, provide an ideal bone regeneration microenvironment. These materials can effectively control infection, suppress bacterial growth, promote the proliferation and differentiation of osteoblasts and accelerate bone tissue regeneration. The degradation properties of these materials allow them to degrade in synchrony with bone repair, avoiding the pain of secondary surgeries for the patient. Additionally, the design of biocomposites can be adjusted according to the needs of individuals, as they have mechanical properties that match those of natural bone and prevent stress shielding, thereby ensuring stability during bone repair. Modern biocomposites can also enhance functionality and effectiveness through the application of nanotechnology and bioactive molecules by reducing the number of surgeries and minimizing the risk of complications. The development and application of these materials also reduce the degree of dependence on donor bones, decrease the incidence of immune rejection reactions and improve patient acceptance and treatment outcomes. Therefore, biocomposites provide a new solution for treating IBDs, overcoming many limitations of traditional methods and offering patients more efficient and safer treatment options.
